# Continuous-flow carbonyl hydrogenation under subatmospheric to atmospheric hydrogen pressure enabled by robust heterogeneous Pt–Fe catalysts

**DOI:** 10.3762/bjoc.22.43

**Published:** 2026-04-10

**Authors:** Hiroyuki Miyamura, Ryosuke Kajiyama, Shun-ya Onozawa, Yoshihiro Kon, Shū Kobayashi

**Affiliations:** 1 Catalytic Chemistry Research Institute, National Institute of Advanced Industrial Science and Technology (AIST), 1-1-1 Higashi, Tsukuba, Ibaraki 305-8565, Japan; 2 Interdisciplinary Research Center for Catalytic Chemistry, National Institute of Advanced Industrial Science and Technology (AIST), 1-1-1 Higashi, Tsukuba, Ibaraki 305-8565, Japan; 3 Department of Chemistry, School of Science, The University of Tokyo, 7-3-1 Hongo, Bunkyo-ku, Tokyo 113-0033, Japan

**Keywords:** bimetallic nanostructure, carbonyl reduction, continuous-flow reaction, heterogeneous catalyst, subatmospheric hydrogen

## Abstract

The reduction of carbonyl compounds, including ketones and aldehydes, to alcohols is a fundamental and important reaction in organic synthesis. One of the most ideal methods is catalytic hydrogenation, however, the hydrogenation of ketones generally requires harsh reaction conditions, such as high temperature and high pressure. We developed a bimetallic Pt–Fe nanoparticle catalyst immobilized on a composite support of dimethylpolysilane and alumina. Both ketones and aldehydes, including highly bulky and sterically hindered substrates, were smoothly hydrogenated using the newly developed catalysts under continuous-flow conditions at room temperature and under subatmospheric to atmospheric hydrogen pressure. High durability of the heterogeneous catalysts was confirmed by a long-term continuous-flow operation. Interestingly, both the combination of metal species and the metal ratio strongly influenced the catalytic performance.

## Introduction

The reduction of carbonyl compounds, ketones and aldehydes to alcohols is a fundamental and important reaction in organic synthesis that can provide valuable chemicals such as functional materials and pharmaceuticals [1–4]. While stoichiometric reagents and catalytic methods are widely developed for this transformation, one of the most ideal methods is heterogeneous catalysis using molecular hydrogen as a reductant, which realizes 100% atom economy [5–12]. In this context, advanced technologies represented by the precise control of the bimetallic structure of a heterogeneous catalyst, mechanochemical hydrogenation, and continuous-flow methods using packed-bed reactors greatly contributed to advancing this transformation [9,11–13].

Recently, continuous-flow organic synthesis has attracted much attention from both academia and industry, because it offers numerous advantages, such as not only a high productivity with limited space but also realizing green sustainable syntheses minimizing required energy and resources. When heterogeneous catalysts are used in a continuous-flow system, the catalyst included in a column allows semi-permanent use without recovery and reuse operations that are usually needed in a batch system. The integration of multiple column reactors also enables multistep continuous production. In addition, an enhanced catalytic performance could be achieved in case of continuous-flow hydrogenation reactions using a gas–liquid–solid catalyst-packed column reactor, in which both a liquid substrate and hydrogen gas can directly interact with the catalytically active sites of the heterogeneous catalyst at an optimal gas/flow ratio [14–29].

Although the hydrogenation of aldehydes is relatively easy to achieve, the hydrogenation of ketones is still challenging, due to their higher steric hindrance and lower electrophilicity. The hydrogenation of ketones often suffers from insufficient reactivity and conversion, even under harsh reaction conditions, such as high temperature and pressurized hydrogen, or when employing advanced technologies [8–9,11–12]. The selective hydrogenation of carbonyl moieties often needs to overcome the problem of overreduction of other functionalities like aromatic systems, and requires additives to suppress this unwanted side-reaction [6,10].

We have developed heterogeneous bimetallic nanoparticle catalysts for the selective hydrogenation of quinizarin to leucoquinizarin under continuous-flow conditions [30]. During our mechanistic study, we unexpectedly discovered that Pt–Fe bimetallic nanoparticles immobilized on a composite support of dimethylpolysilane (DMPSi) and alumina (Pt‒Fe/DMPSi–Al_2_O_3_) exhibited extraordinary catalytic performance for the selective hydrogenation of ketones under continuous-flow and atmospheric pressure hydrogen conditions. In this article, we report the development of a continuous-flow carbonyl reduction system that achieves high performance and a wide substrate scope under atmospheric or subatmospheric hydrogen pressure and ambient temperature using Pt–Fe bimetallic heterogeneous catalysts.

## Results and Discussion

### Catalyst preparation

First, we prepared Pt‒Fe/DMPSi‒Al_2_O_3_ with different Fe/Pt ratios by the simultaneous reduction of Pt and Fe salts in a solution containing dissolved sodium borohydride (NaBH_4_) and suspended DMPSi [15,30–32]. After DMPSi stabilized the resulting bimetallic nanoparticles, Al_2_O_3_ was added to the mixture, followed by methanol. The resulting solid catalyst was collected by filtration, washed with solvents, and heated twice. Si‒Si bonds in DMPSi were partially oxidized to form Si‒O‒Si bonds during the preparation process, and they became cross-linked to Al_2_O_3_ to form a stabilized composite support [32]. A plausible scenario for the formation of the bimetallic structure of Pt‒Fe nanoparticles during the catalyst preparation would be the same as that for the formation of Pt–Ni bimetallic structures, and it involves the following steps [33]. Na_2_PtCl_6_∙6H_2_O might be reduced by NaBH_4_ faster than FeCl_2_ in the solution phase, and the generated Pt(0) nanoparticles are stabilized by DMPSi surface. The reduction of FeCl_2_, which is usually more difficult to achieve can be catalyzed on the surface of Pt(0) nanoparticles and the formed Fe species are deposited and grown from the existing Pt nanoparticles. Therefore, a bimetallic structure of Pt and Fe is generated, and both elements are observed with similar distributions by STEM‒EDS mapping analysis (Figure S1, Supporting Information File 1). The Pt nanoparticles are isolated by the surrounding Fe species, which prevents aggregation and preserves their small size (2–5 nm). The valence of the Pt and Fe species was found to be Pt(0) and Fe(II) in the catalyst by XPS analysis [30]. The valence of Fe species may have been 0 during the reduction step of the catalyst preparation procedure. However, the Fe species can easily oxidize to Fe(II) during the filtration and heating processes, resulting in the formation of a bimetallic structure comprising Pt(0) and Fe(II). Pt‒Au, Pt‒Co, and Pt‒Ni bimetallic catalysts immobilized on DMPSi‒Al_2_O_3_ were also prepared by the same procedure.

### Comparison of the catalysts in the continuous-flow hydrogenation of a ketone

**Scheme 1:**
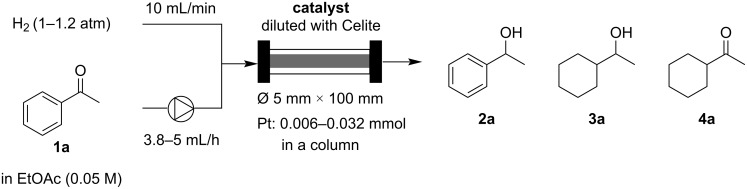
Hydrogenation of a ketone under continuous-flow conditions.

We compared the catalytic performance of the newly prepared bimetallic catalysts to that of commercially available heterogeneous Pt catalysts in the continuous-flow hydrogenation of acetophenone (**1a**) at room temperature. The heterogeneous catalysts were packed into a column with Celite, with the molar amount of Pt adjusted to 0.006 mmol within the column. Both the solution of acetophenone (**1a**) in ethyl acetate (EtOAc) and hydrogen gas were simultaneously passed through the catalyst-packed column without backpressure control at room temperature (Scheme 1). The powder-like catalysts were packed in a glass column equipped with glass wool filters at both ends of the column. The hydrogen gas and liquid substrate were introduced through a double-layered column head, in which the substrate solution is directly injected to the glass wool filter of the column by a PTFE tube and the hydrogen gas is supplied by an outer layer surrounding the PTFE tube (Figure S2, Supporting Information File 1). Thus, the hydrogen gas and liquid substrate can be mixed at the glass wool filter which is located at the top of the catalyst column, and the well mixed gas–liquid is supplied to the catalyst region in the column. No backpressure controller is installed, and the outlet of the column is directly released to atmosphere. When the flow rates of hydrogen gas and substrate solution were 10 mL/min and 3.8‒5 mL/h, respectively, the pressure at the inlet of the column was almost 1.2 atm in this case (0.2 atm pressure loss). The yields of 1-phenetylalcohol (**2a**) and by-products **3a** and **4a** under these reaction conditions for each catalyst are summarized in Table 1. The commercially available catalysts (Pt/C, Pt/SiO_2_, Pt/Al_2_O_3_) and Pt/DMPSi‒Al_2_O_3_ showed poor to moderate reactivity, and the hydrogenation of the aromatic moiety proceeded as well to give by-products **3a** and **4a** (Table 1, entries 1‒4). Pt‒Au and Pt‒Ni bimetallic catalysts immobilized on DMPSi‒Al_2_O_3_ also showed moderate reactivity, accompanied by the formation of by-products (Table 1, entries 5 and 6). Interestingly, Pt‒Co and Pt‒Fe bimetallic catalysts immobilized on DMPSi‒Al_2_O_3_ showed high activity with minimal by-product formation (Table 1, entries 7‒11). Especially, Pt‒Fe/DMPSi‒Al_2_O_3_ (Fe/Pt = 0.62‒2.3) demonstrated excellent catalytic performance under the continuous-flow conditions giving the desired product with 95% to >99% yield and almost no by-product formation (Table 1, entries 8–10). However, the reactivity of the Pt‒Fe/DMPSi‒Al_2_O_3_ catalyst decreased when the Fe/Pt ratio was excessively high (Table 1, entry 11). We also prepared a Pt–Fe/Al_2_O_3_ catalyst without the use of DMPSi during catalyst synthesis. However, Pt–Fe/Al_2_O_3_ catalyst showed very low catalytic activity compared to Pt‒Fe/DMPSi‒Al_2_O_3_ (Table 1, entry 12). This suggested that both the bimetallic structure of Pt–Fe and DMPSi in the support are required for the high catalytic performance. We also compared the catalytic performance in detail by varying the flow rate of the substrate solution in the flow system, and the catalytic turnover frequency (TOF) values of all catalysts tested at each flow rate are summarized in Supporting Information File 1 (Tables S1‒11 and Figures S3 and S4). The Pt‒Fe/DMPSi‒Al_2_O_3_ (Fe/Pt = 0.62) catalyst showed the highest TOF of >50 h^−1^ in this investigation, although overreduction occurred to some extent with this catalyst (Table 1, entry 8). Both the combination of metal species in bimetallic catalysts and the ratio of metals were important to optimize the catalytic performance with respect to activity and selectivity.

**Table 1:** Comparison of catalyst performance in the hydrogenation of a ketone under continuous-flow conditions.

Entry	Catalyst	Fe/Pt ratio	Yield (%)^a^			
			
			**2a**	**3a**	**4a**	**1a**

1	Pt/C	–	16	2	10	72
2	Pt/SiO_2_	–	2	0	3	95
3	Pt/Al_2_O_3_	–	36	5	13	47
4	Pt/DMPSi‒Al_2_O_3_	–	9	1	7	84
5	Pt-Au/DMPSi‒Al_2_O_3_	–	38	3	12	47
6	Pt-Ni/DMPSi‒Al_2_O_3_	–	32	2	0	66
7	Pt-Co/DMPSi‒Al_2_O_3_	–	81	2	0	17
8	Pt‒Fe/DMPSi‒Al_2_O_3_	0.62	95	5	0	0
9	Pt‒Fe/DMPSi‒Al_2_O_3_	1.4	97	2	0	1
10	Pt‒Fe/DMPSi‒Al_2_O_3_	2.3	>99	0	0	0
11	Pt‒Fe/DMPSi‒Al_2_O_3_	4.4	74	0	0	26
12	Pt-Fe/Al_2_O_3_	0.73	11	trace	1	88

^a^Yield was determined by GC analysis using decane as an internal standard.

The binding energies of Pt 4d in Pt–Fe/DMPSi‒Al_2_O_3_ (332, 315 eV) are lower than those of Pt/DMPSi‒Al_2_O_3_ (333, 316 eV) and Pt–Ni/DMPSi‒Al_2_O_3_ (334, 317 eV) [30]. Thus, the Pt species in Pt–Fe/DMPSi‒Al_2_O_3_ is the most electronically negative among the catalysts, which leads to a stronger reducing ability after adsorption of hydrogen on the surface of the catalyst. Therefore, the more demanding carbonyl hydrogenation is enabled by the stronger reducing ability of the Pt-Fe/DMPSi‒Al_2_O_3_ catalyst. We also investigated the effect of solvent and ethyl acetate (EtOAc) was found to be the best among the solvents tested (EtOAc, toluene, methylcyclohexane, tetrahydrofuran, and methanol) (see Supporting Information File 1, Figure S5). The hydroxy moiety has a stronger interaction with catalytically active sites at the surface of the heterogeneous catalyst, and smooth desorption of the alcohol product is key for a high catalytic turnover. The polar solvent, EtOAc, facilitates the desorption of the formed alcohol by stabilizing it in the solution phase, leading to the smooth product desorption and following substrate adsorption. Although methanol and THF are also polar solvents, they can be strongly adsorbed on the catalytically active sites of the catalyst leading to a poisoning effect by these solvents and disturbing the catalytic turnover.

### Substrate scope

**Scheme 2:**
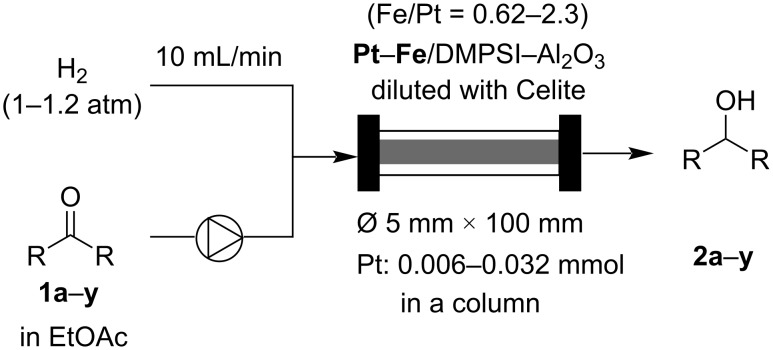
Continuous-flow hydrogenation of carbonyl compounds.

**Figure 1:**
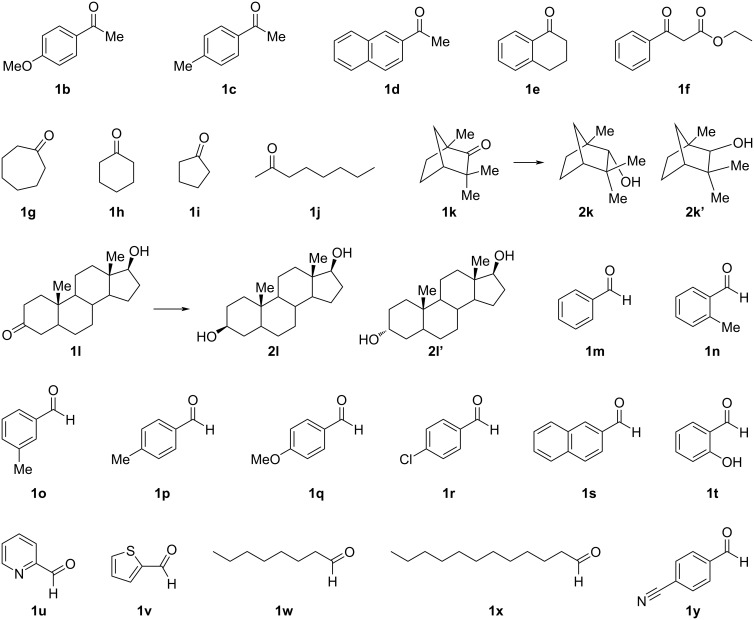
List of substrates.

We also investigated the substrate scope under continuous-flow conditions using the best catalyst, Pt‒Fe/DMPSi‒Al_2_O_3_ (Fe/Pt = 0.62‒2.3) (Scheme 2, Figure 1, and Table 2). 4’-Methoxyacetophenone (**1b**) and 4’-methylacetophenone (**1c**) were quantitatively hydrogenated to the corresponding alcohols **2b** and **2c** under continuous-flow conditions at room temperature (Table 2, entries 1 and 2). The bulky substrate 2-acetylnaphthalene (**1d**) was converted to the corresponding alcohol **2d** in 89% yield under continuous-flow conditions at room temperature (Table 2, entry 3). The desired product **2d** was obtained quantitatively at 50 °C (Table 2, entry 4). It is noteworthy that an analytically pure compound (confirmed by ^1^H and ^13^C NMR) was isolated by simple evaporation of the solvent (Table 2, entry 4).

**Table 2:** Substrate scope under the continuous-flow hydrogenation of carbonyl compounds.

Entry	Substrate	Temperature	Yield (%)

1	**1b**	rt	>99^a^
2	**1c**	rt	>99^a^
3	**1d**	rt	89^a^
4	**1d**	50 °C	>99^b^
5	**1e**	rt	88^a^
6	**1f**	rt	>99^b^
7	**1g**	rt	95^a^
8	**1h**	rt	>99^a^
9	**1i**	rt	98^a^
10	**1j**	50 °C	>99^a^
11	**1k**	80 °C	74^a,c^
12	**1l**	50 °C	>99^b,d^
13	**1m**	rt	>99^a^
14	**1n**	rt	>99^a^
15	**1o**	rt	>99^a^
16	**1p**	rt	>99^a^
17	**1q**	rt	>99^a^
18	**1r**	rt	98^a^
19	**1s**	rt	>99^a^, >99^b^
20	**1t**	rt	>99^a^, >99^b^
21	**1u**	rt	91^a^
22	**1v**	50 °C	61^a^
23	**1w**	rt	99^a^
24	**1x**	rt	>99^b^
25	**1y**	30 °C	95^a^

^a^Yield was determined by GC analysis using decane as an internal standard. ^b^Analytically pure isolated compound was obtained after removal of the solvent from the eluted solution from the continuous-flow reactor. ^c^The ratio of **2k**:**2k’** was 66:34. ^d^A mixture of diastereomers was isolated, and the ratio of **2l**:**2l’** was determined to be 74:26 by ^1^H NMR analysis. rt = room temperature.

Tetralone (**1e**) was also hydrogenated smoothly to afford the corresponding alcohol **2e** in 88% yield at room temperature (Table 2, entry 5). Ethyl benzoylacetate (**1f**) was converted to the corresponding alcohol **2f** quantitatively while retaining the ester moiety, and the product was isolated by simple evaporation of the solvent from the collected fraction eluted from the flow reactor (Table 2, entry 6). Cyclic ketones **1g**, **1h**, and **1i** and an aliphatic ketone **1j** were also hydrogenated in excellent yields at room temperature (Table 2, entries 7–10). Fenchone (**1k**) has a fused bicyclic skeleton with a carbonyl moiety positioned between two quaternary carbons. Therefore, its carbonyl moiety is sterically shielded and its hydrogenation is highly challenging. A mixture of two diastereomers **2k** and **2k’** was obtained in 74% yield (**2k**:**2k’** = 66:34) at 80 °C under the continuous-flow conditions (Table 2, entry 11). The high catalytic performance of Pt‒Fe/DMPSi‒Al_2_O_3_ enabled the hydrogenation of substrate **1k** even under almost atmospheric pressure hydrogen conditions. Stanolone (**1l**) is also a bulky ketone compound with a steroid skeleton, and its hydrogenation is challenging. The continuous-flow hydrogenation of **1l** proceeded quantitatively to afford two diastereomers in a 74:26 ratio even at room temperature (Table 2, entry 12).

Next, we investigated the hydrogenation of aldehydes. Benzaldehyde, 2-, 3-, and 4-methylbenzaldehyde **1m**, **1n**, **1o**, and **1p** were quantitatively hydrogenated to the corresponding alcohols without over-reduction (Table 2, entries 13–16). 4-Methoxybenzaldehyde (**1q**) was also hydrogenated quantitatively under the continuous-flow conditions (Table 2, entry 17). 4-Chlorobenzaldehyde (**1r**) was converted to 4-chlorobenzylalcohol quantitatively while retaining the chloride moiety (Table 2, entry 18). The catalytic turnover frequencies (TOFs) of these aldehydes under continuous-flow conditions reached 180 h^−1^ (**1m**: 180 h^−1^, **1n**: 104 h^−1^, **1o**: 144 h^−1^, **1p**: 130 h^−1^, **1q**: 88 h^−1^, **1r**: 127 h^−1^; see Supporting Information File 1 for details). 2-Naphthaldehyde (**1s**) and salicylaldehyde (**1t**) were hydrogenated quantitatively, and analytically pure products were isolated by simple evaporation of the collected fraction eluted from the flow reactor (Table 2, entries 19 and 20). Heterocyclic aldehydes **1u** and **1v** were also applicable, while the conversion of **1v** was moderate, probably because of the poisoning effect of the sulfur atom in the compound (Table 2, entries 21 and 22). Aliphatic aldehydes **1w** and **1x** were also hydrogenated quantitatively under atmospheric hydrogen and room temperature conditions (Table 2, entries 23 and 24), and an analytically pure alcohol was isolated by simple evaporation of the collected fraction eluted from the flow reactor (Table 2, entry 24). Remarkably, when 4-cyanobenzaldehyde (**1y**) was used as the substrate, selective hydrogenation of the carbonyl moiety proceeded to afford 4-cyanobenzylalcohol (**2y**) in 95% yield, preserving the nitrile moiety which is typically hydrogenated under similar conditions (Table 2, entry 25).

### Durability of the catalyst under the continuous-flow conditions

**Figure 2:**
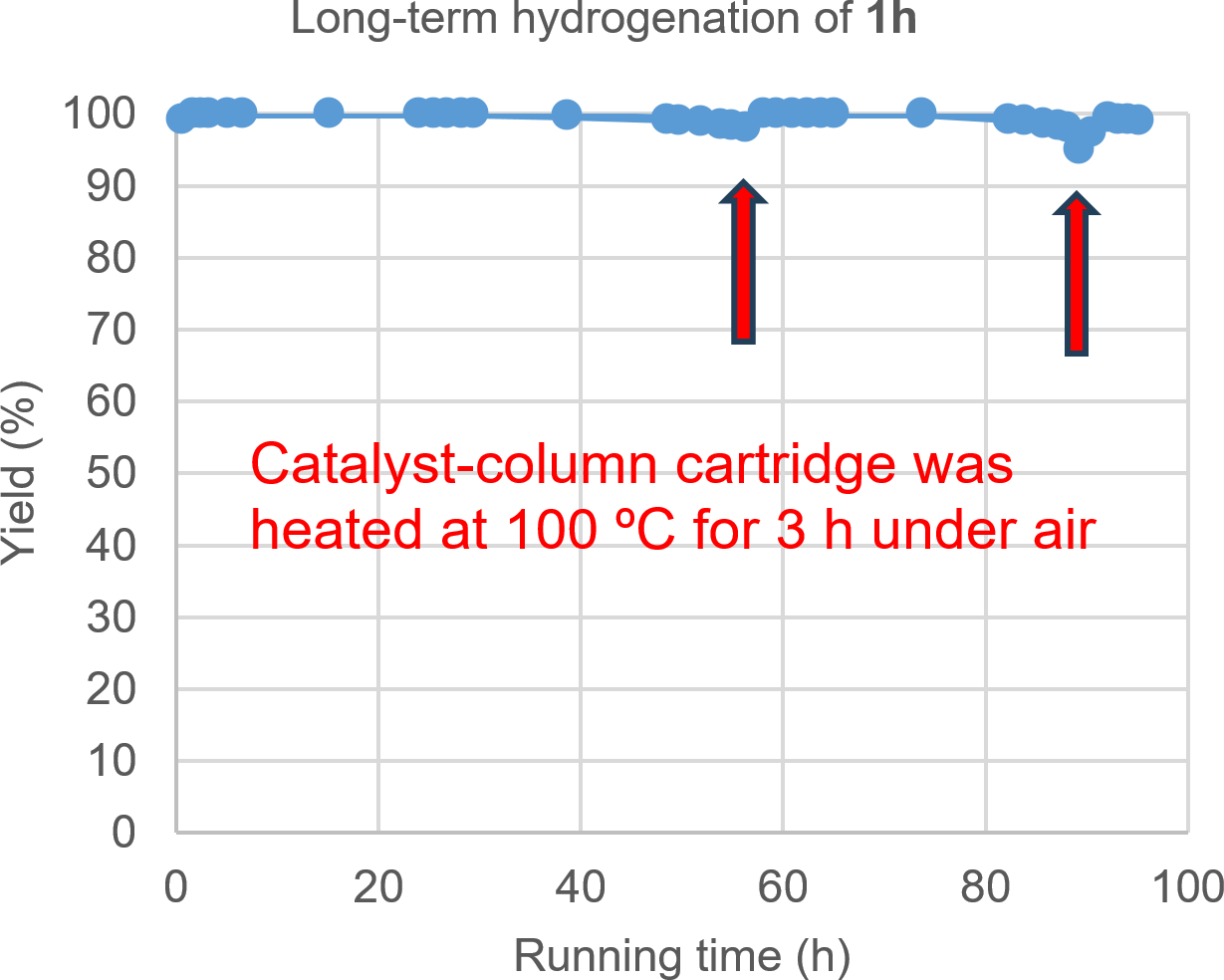
Continuous-flow hydrogenation of cyclohexanone **1h**. Conditions: solution of **1h** in EtOAc (0.06 M) with decane as an internal standard; substrate flow rate, 7.2 mL h^−1^; hydrogen flow rate, 10 mL min^−1^; amount of Pt in the column, 0.006 mmol; reaction temperature, 30 °C.

We investigated the durability of Pt‒Fe/DMPSi‒Al_2_O_3_ under continuous-flow hydrogenation conditions at steady state during a long-period run (Figure 2). Quantitative conversion of cyclohexanone (**1h**) was maintained over the initial 50 h; however, a small amount of **1h** remained after 54 h of continuous running. Then, the supply of substrate and hydrogen was stopped, and the catalyst column was heated at 100 °C for 3 h to reactivate the catalyst. The continuous-flow hydrogenation was restarted after the column had cooled to ambient temperature. Catalytic activity was successfully restored, and quantitative conversion was maintained for a further 24 h. A small amount of **1h** remained again, and the catalyst regeneration process was repeated. Finally, the catalytic activity was revived again, and quantitative conversion was observed over 90 h of total running. Water as contaminant in EtOAc or organic compounds strongly adsorbed on the catalyst’s surface may have led to its deactivation; however, they could be removed by simple heating. We demonstrated the robustness of Pt‒Fe/DMPSi‒Al_2_O_3_ under continuous-flow hydrogenation conditions and showed that a simple heat treatment process for regenerating the catalytic activity is adaptable for practical use of this continuous-flow system.

### Continuous-flow hydrogenation of carbonyl compounds under subatmospheric pressure of hydrogen

**Scheme 3:**
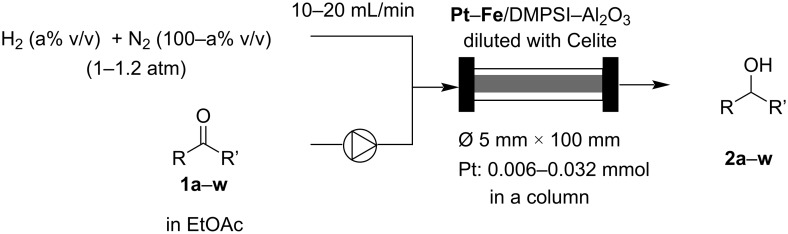
Continuous-flow hydrogenation of carbonyl compounds under subatmospheric partial pressure of hydrogen.

**Figure 3:**
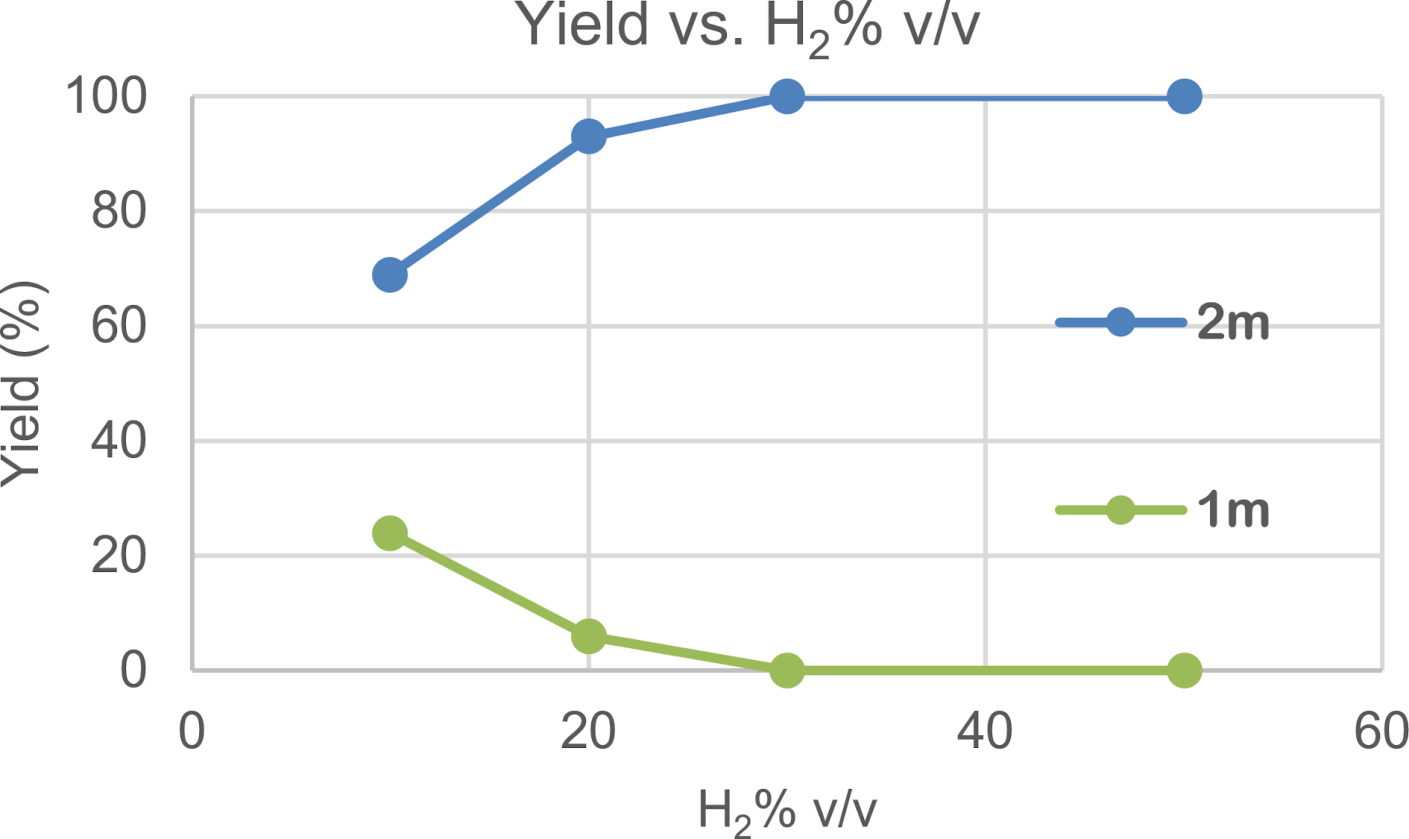
Continuous-flow hydrogenation of benzaldehyde **1m** by varying the H_2_/N_2_ v/v ratio. Conditions: solution of **1m** in EtOAc (0.06 M) with decane as an internal standard; substrate flow rate, 10 mL h^−1^; H_2_ + N_2_ mixed-gas flow rate, 20 mL min^−1^; amount of Pt in the column, 0.032 mmol; reaction temperature, room temperature.

Next, we investigated whether continuous-flow hydrogenation could proceed under subatmospheric partial pressure of hydrogen gas. The use of subatmospheric partial pressure of hydrogen gas is highly desired from the viewpoint of safety, as well as for utilizing green hydrogen produced by sustainable energy, because purifying and pressurizing hydrogen gas consumes large amounts of energy comparable to the energy required to generate green hydrogen [34]. We set up a continuous-flow system in which a mixture of H_2_ and N_2_ was introduced by two individually regulated mass-flow controllers (Scheme 3). First, the continuous-flow hydrogenation of benzaldehyde (**1m**) was investigated by varying the H_2_/N_2_ v/v ratio. Yields of **2m** and **1m** at the steady state of continuous-flow hydrogenation with different H_2_/N_2_ v/v ratios are plotted in Figure 3. Quantitative conversion of **1m** was observed with 50% and 30% v/v of hydrogen and product **2m** was obtained in 93% yield even at 20% v/v of hydrogen; the partial pressure of hydrogen in the catalyst column was 0.2‒0.24 atm (Table 3, entry 4). A 69% yield of **2m** was obtained with just 10% v/v of hydrogen. Acetophenone (**1a**) was successfully hydrogenated with 20% v/v hydrogen to afford the desired product quantitatively (Table 3, entry 1). A cyclic ketone **1i** was converted to **2i** in 84% yield with 10% v/v hydrogen and in 97% yield with 20% v/v hydrogen (Table 3, entries 2 and 3). An aliphatic aldehyde **1w** was also hydrogenated to give the product in 97% yield with 20% v/v hydrogen (Table 3, entry 5). These results were compared with other representative catalytic systems for the hydrogenation of carbonyl compounds (see Supporting Information File 1, Table S12), and the high catalytic performance of Pt‒Fe/DMPSi‒Al_2_O_3_ even under subatmospheric partial pressure of hydrogen was highlighted [8–12].

**Table 3:** Substrate scope using subatmospheric partial pressure of hydrogen.

Entry	Substrate	H_2_% v/v	Total gas flow rate (H_2_ + N_2_) (mL min^−1^)	Yield (%)^a^

1	**1a**	20	20	>99
2	**1i**	10	20	84
3	**1i**	20	20	97
4	**1m**	20	20	93
5	**1w**	20	10	97

^a^Yield was determined by GC analysis using decane as an internal standard.

## Conclusion

In summary, we developed bimetallic Pt‒Fe/DMPSi‒Al_2_O_3_ as a powerful heterogeneous catalyst for the hydrogenation of carbonyl compounds under continuous-flow and ambient conditions. Both ketones and aldehydes, including highly bulky and sterically hindered substrates, were smoothly hydrogenated at room temperature under subatmospheric to atmospheric hydrogen pressure with a wide substrate scope. The high durability of the heterogeneous catalyst was confirmed by a long-term continuous-flow reaction, and the catalytic activity was revived by a simple operation even if the catalyst became slightly deactivated. Interestingly, both the combination of metal species in the catalysts and the ratio of metals had a great impact on catalytic performance. The newly developed continuous-flow hydrogenation reaction under mild conditions is adaptable to sustainable chemical synthesis, minimizing energy consumption and enabling the use of green hydrogen. In addition, the newly obtained insights regarding the relationships among the Fe/Pt ratios in the catalysts, the bimetallic structure, and the resulting catalytic performance for selective hydrogenation will guide the future development of heterogeneous catalysts.

## Supporting Information

File 1 Additional experimental details, materials, and methods including photographs of reactor systems, STEM-EDS images and XPS spectra of heterogeneous catalysts, and copies of ^1^H and ^13^C NMR spectra for isolated compounds.

## Data Availability

All data that supports the findings of this study is available in the published article and/or the supporting information of this article.

## References

[1] Hudlicky M (1984). Reductions in Organic Chemistry.

[2] Trost B M, Fleming I (1991). Reduction. Comprehensive Organic Synthesis.

[3] Nishimura S (2001). Handbook of Heterogeneous Catalytic Hydrogenation for Organic Synthesis.

[4] de Vries J G, Elsevier C J (2007). The Handbook of Homogeneous Hydrogenation.

[5] Mäki-Arvela P, Hájek J, Salmi T, Murzin D Y (2005). Appl Catal, A.

[6] Fujiwara Y, Iwasaki Y, Maegawa T, Monguchi Y, Sajiki H (2011). ChemCatChem.

[7] Cano I, Chapman A M, Urakawa A, van Leeuwen P W N M (2014). J Am Chem Soc.

[8] Tan J, Cui J, Cui X, Deng T, Li X, Zhu Y, Li Y (2015). ACS Catal.

[9] Osako T, Torii K, Hirata S, Uozumi Y (2017). ACS Catal.

[10] Duraczyńska D, Serwicka E M, Drelinkiewicz A, Socha R P, Zimowska M, Lityńska-Dobrzyńska L, Bukowska A (2019). Mol Catal.

[11] Matsuda S, Masuda S, Takano S, Ichikuni N, Tsukuda T (2021). ACS Catal.

[12] Mayer M, Wohlgemuth M, Salomé Straub A, Grätz S, Borchardt L (2025). Angew Chem, Int Ed.

[13] Zanette T, García-Zaragoza A, Mazarío J, Santiago Martinez J, Chaudret B, Cerezo-Navarrete C, Oña-Burgos P (2025). Green Chem.

[14] Irfan M, Glasnov T N, Kappe C O (2011). ChemSusChem.

[15] Miyamura H, Suzuki A, Yasukawa T, Kobayashi S (2018). J Am Chem Soc.

[16] Cai B, Cheo H W, Liu T, Wu J (2021). Angew Chem, Int Ed.

[17] Asano S, Miyamura H, Matsushita M, Kudo S, Kobayashi S, Hayashi J-i (2024). J Flow Chem.

[18] Laporte A A H, Masson T M, Zondag S D A, Noël T (2024). Angew Chem, Int Ed.

[19] Yoshida J-i, Saito K, Nokami T, Nagaki A (2011). Synlett.

[20] Hessel V, Kralisch D, Kockmann N, Noël T, Wang Q (2013). ChemSusChem.

[21] Wiles C, Watts P (2014). Green Chem.

[22] Gutmann B, Cantillo D, Kappe C O (2015). Angew Chem, Int Ed.

[23] Ley S V, Fitzpatrick D E, Myers R M, Battilocchio C, Ingham R J (2015). Angew Chem, Int Ed.

[24] Kobayashi S (2016). Chem – Asian J.

[25] Plutschack M B, Pieber B, Gilmore K, Seeberger P H (2017). Chem Rev.

[26] Rogers L, Jensen K F (2019). Green Chem.

[27] Weeranoppanant N (2019). React Chem Eng.

[28] Ferlin F, Lanari D, Vaccaro L (2020). Green Chem.

[29] Capaldo L, Wen Z, Noël T (2023). Chem Sci.

[30] Miyamura H, Sharma A, Takata M, Kajiyama R, Kobayashi S, Kon Y (2024). ACS Catal.

[31] Miyamura H, Tobita F, Suzuki A, Kobayashi S (2019). Angew Chem, Int Ed.

[32] Kobayashi S, Okumura M, Akatsuka Y, Miyamura H, Ueno M, Oyamada H (2015). ChemCatChem.

[33] Miyamura H, Kajiyama R, Kon Y (2025). Top Catal.

[34] Segovia-Hernández J G, Hernández S, Cossío-Vargas E, Juarez-García M, Sánchez-Ramírez E (2025). RSC Sustainability.

