# Supplementary material for: Continuous-flow carbonyl hydrogenation under subatmospheric to atmospheric hydrogen pressure enabled by robust heterogeneous Pt–Fe catalysts
Source: Beilstein J Org Chem. 2026 Apr 10;22:575–82. doi: 10.3762/bjoc.22.43 (PMC13071934; doi:10.3762/bjoc.22.43)
Supplement: File 1 — Additional experimental details, materials, and methods including photographs of reactor systems, STEM-EDS images and XPS spectra of heterogeneous catalysts, and copies of 1H and 13C NMR spectra for isolated compounds. [file Beilstein_J_Org_Chem-22-575-s001.pdf]

BEILSTEIN JOURNAL OF ORGANIC CHEMISTRY

Supporting Information

for

Continuous-flow carbonyl hydrogenation under subatmospheric to atmospheric hydrogen pressure enabled by robust heterogeneous Pt–Fe catalysts

Hiroyuki Miyamura, Ryosuke Kajiyama, Shun-ya Onozawa, Yoshihiro Kon and Shū Kobayashi

Beilstein J. Org. Chem. **2026,** *22,* 575–582. doi:10.3762/bjoc.22.43

Additional experimental details, materials, and methods including photographs of reactor systems, STEM-EDS images and XPS spectra of heterogeneous catalysts, and copies of ^{1}H and ^{13}C NMR spectra for isolated compounds

License and Terms: This is a supporting information file under the terms of the Creative Commons Attribution License (https://creativecommons.org/licenses/by/4.0). Please note that the reuse, redistribution and reproduction in particular requires that the author(s) and source are credited and that individual graphics may be subject to special legal provisions.
The license is subject to the *Beilstein Journal of Organic Chemistry* terms and conditions: (https://www.beilstein-journals.org/bjoc/terms)

Table of contents

General remarks S2

Preparation of catalysts S4

M/DMPSi-Al_2O_3 .... S4

Hydrogenation in continuous-flow system S6

Reaction apparatus for hydrogenation S6

Procedure for hydrogenation of carbonyl compounds under continuous-flow conditions S7

Detailed results of substrate scope varying MHSV or isolation of products S15

Comparison of heterogeneous catalysts for hydrogenation of carbonyl compounds S31

^1H and ^{13}C NMR spectra of isolated compounds S32

1-(2-Naphtyl)ethanol (**2d**) S32

Ethyl 3-hydroxy-3-phenylpropanate (**2f**) S33

Naphthalen-2-ylmethanol (**2s**) S34

2-(Hydroxymethyl)phenol (**2t**) S35

1-Dodecanol (**2x**) S36

2l + **2l'** S37

References S38

General remarks

- NMR spectra were recorded with Bruker Avance NEO 400 MHz spectrometer operating at 400 MHz (^1H) and 101 MHz (^{13}C) respectively. Chemical shifts were recorded in parts per million (ppm), from relative to internal references of the $CDCl_3$, defined at 7.24 ppm (^1H NMR) and 77.0 ppm (^{13}C NMR) otherwise noted.
- GC analysis was performed on a Shimadzu GC-2030 apparatus with FID detector (column = Shimadzu SH-Polarwax, 0.25 μm, 60 m; N_2 gas pressure: 274.1 kPa, total flow: 144.3 mL/min, column flow: 3.01 mL/min, linear velocity: 42.5 cm/s, sprit ratio: 46.0, injection temperature: 250 °C, detector temperature: 280 °C, H_2 flow = 32.0 mL/min, air flow = 200 mL/min). Decane was used as internal standard for quantitative analysis.
- Inductively coupled plasma–atomic emission spectrometry (ICP–AES) analysis was performed on Horiba ULTIMA2 equipment.
- Aqua regia for ICP sample preparation was made by mixing concentrated hydrochloric acid and concentrated nitric acid in a volume of 3:1.
- Energy-dispersive X-ray spectroscopy/scanning transmission electron microscopes (STEM/EDS) images were obtained using a FEI JTECNAI OSIRIS instrument operated at 200 kV. All STEM specimens were prepared by placing a drop of the solution on carbon-coated copper grids and allowed to dry in air (without staining).
- X-ray photoelectron spectroscopy (XPS) was conducted by KRATOS ULTRA2 (Shimadzu). Binding energy was calibrated using internal carbon peak (C1s = 285.0 eV).
- $NaBH_4$ was purchased from Fujifilm Wako Pure Chemical and it was stored and handled in a glove box.
- Organic reagents were used as purchased, or purified by distillation or recrystallization following the normal procedures if it is needed.

- Purchased from Fujifilm Wako Pure Chemical: **1b**, **2b**, **2e**,**1g**, **1h**, **2h**, **1i**, **2i**, **1j**, **2j**, **2m**, **1g**, **2g**, **1r**, **2u**, **1w**, **1x**. Purchased from TCI: **1a**, **1c**, **2c**, **1d**, **2d**, **1e**, **1f**, **2f**, **2g**, **1k**, **2k**, **2k'**, **1l**, **1m**, **1n**, **2n**, **1p**, **2p**, **1o**, **2o**, **2r**, **1s**, **2s**, **1t**, **2t**, **1u**, **1v**, **2v**, **2w**, **2x**. Purchased from Alfa: **2b**, purchased from Aldrich: **1v**. Purchased from BLD Pharmatech: **2v**

- Solvents and Celite® were purchased from Fujifilm Wako Pure Chemical. Deactivated glass wool (24324) was purchased from RESTEK.

- Pt/Al_2O_3 (5 wt %) (Fujifilm Wako Pure Chemical), Pt/SiO_2 (5 wt %)(Strem), Pt/C (3 wt %)(Fujifilm Wako Pure Chemical), were used as commercially available catalysts.

- $Na_2PtCl_6 \cdot 6H_2O$ was purchased from Aldrich. Pt black, PtO_2, $PtCl_2$, $FeCl_2$, Fe_2O_3, Fe_3O_4, Fe(0), $NiCl_2 \cdot 6H_2O$, and $CoCl_2 \cdot 6H_2O$ were purchased from Fujifilm Wako Pure Chemical

- Polisilane (DMPSi) was purchased from NIPPON SODA CO. LTD, and basic Al_2O_3 was purchased from Merck. Pt/DMPSi-Al_2O_3 and Pt-Au/DMPSi-Al_2O_3 were prepared, following a method described in the literature [1].

Preparation of catalysts

M/DMPSi–Al_2O_3

M^1–M^2/DMPSi-Al_2O_3 catalysts were prepared, following procedures that have been reported in our previous article.[1] Pt–Fe/DMPSi-Al_2O_3 catalysts with different Pt/Fe ratios were also prepared in a manner analogous to previously reported article.[1]

A representative procedure is given below.

A procedure for the preparation of Pt–Fe/DMPSi–Al_2O_3:

To a suspension of polydimethylsilane (0.25 g) in THF (4 mL) was added $NaBH_4$ (81.4 mg) dissolved in diglyme (3 mL) at room temperature. $Na_2PtCl_6{\cdot}6H_2O$ (67.4 mg) and $FeCl_2$ (15.8 mg) dissolved in THF (2 mL) was added dropwise to the solution at room temperature, and the reaction mixture was stirred at room temperature for 3 h. Then, basic alumina (1.25 g) was added to the mixture, which was then stirred at room temperature for 24 h. MeOH (200 mL) was added slowly to induce co-acervation, and the mixture was stirred at room temperature overnight. The resulting solid was collected by filtration and subsequently washed with a large excess of water until no brown solution came out from the filtrate. Afterward, it was washed with 100 mL of acetone, and the catalyst was kept wet with acetone to prevent the catalyst from burning. The collected solid was then dried in a vacuum at 100 °C for 3 h. Subsequently, the solid was heated to 150 °C under vacuum for 5 h. The solid was cooled under an argon atmosphere, after which the solid was washed with acetone (50 mL), water (50 mL), and CH_2Cl_2 (50 mL) then dried in vacuo at 150 °C to afford the Pt–Fe/DMPSi–Al_2O_3 (1.5 g, Pt: 0.012 mmol/g Fe: 0.020 mmol/g).

EDS mapping of Pt–Fe/DMPSi–Al_2O_3 (Fe/Pt = 1.4):

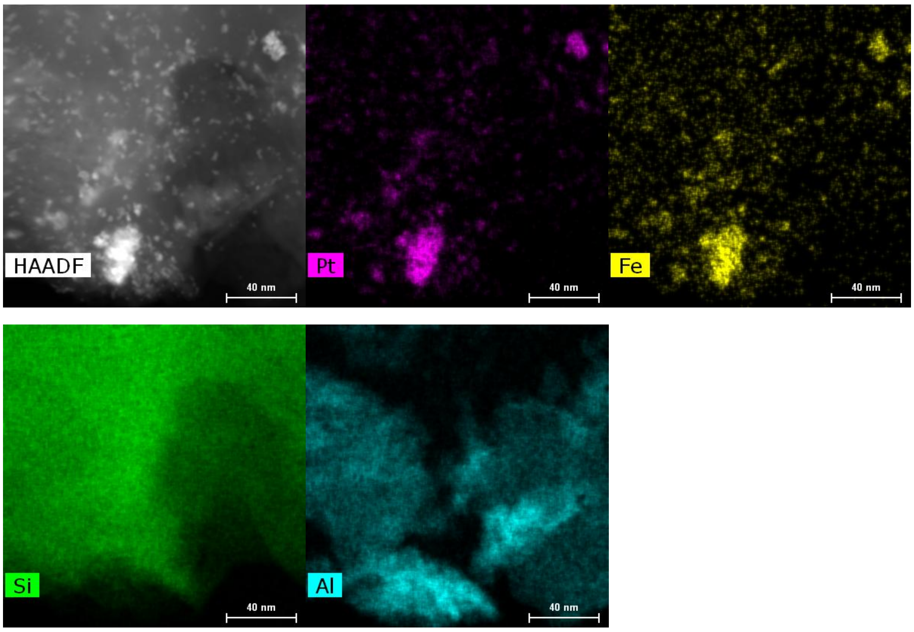

Figure S1. STEM-EDS mapping of Pt–Fe/DMPSi–Al_2O_3

Procedure to determine catalyst loading using ICP

Approximately 2–5 mg of catalyst was measured (with the amount measured being recorded) and placed in a test tube to which 1 mL of sulfuric acid was then added. The test tube was heated to 180 °C, and nitric acid was added dropwise until all solids had dissolved and no more brown fumes were observed. The mixture was cooled to room temperature and aqua regia (1 mL) was added slowly. The mixture was then treated by sonication until black precipitates were completely dissolved. The resulting mixture was brought up to a final volume of 50 mL with water in a volumetric flask, and the resulting solution was subjected to ICP analysis.

Hydrogenation in continuous-flow system

Reaction apparatus for hydrogenation

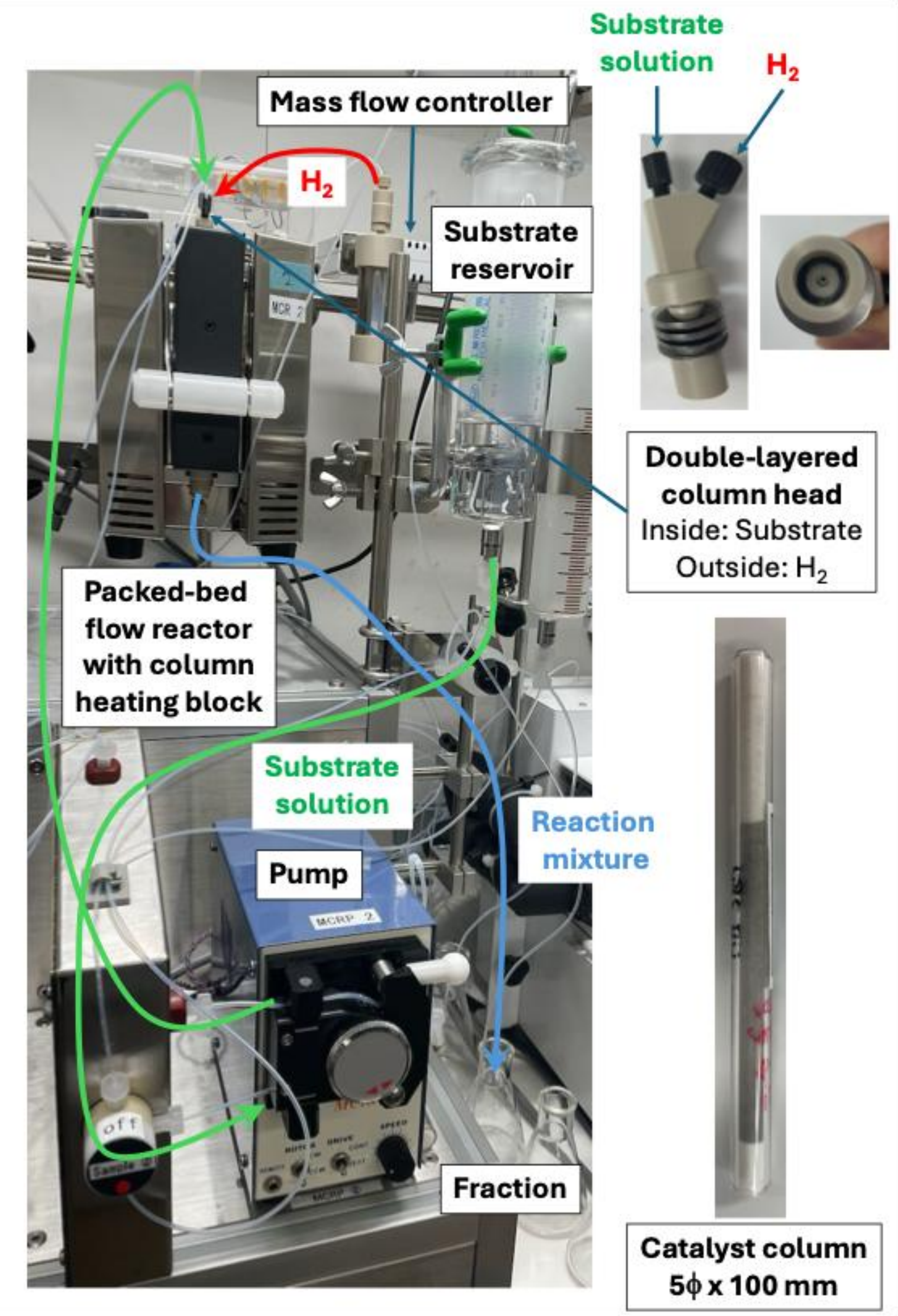

Figure S2. Photograph of flow reactor, double-layered colum head and catalyst column

Continuous-flow reactions were conducted using an EYELA MCR-1000 column flow reactor (SynpleFlow™), a Minatoconcept MCRP204 peristaltic pump unit for introducing a liquid substrate, an EYELA MFC-11GU mass flow controller for introducing hydrogen gas (1–1.2 atm), and an EYELA MFC-1000 system that operates four individual mass flow controllers simultaneously for introducing mixed gas of H_2 and N_2. Hydrogen gas was supplied by a PEAK Precision Hydrogen SL200 hydrogen gas generator. A 1/16-inch PTFE tube was used for all liquid and gas lines. Both gas and liquid were directed downward through a column head featuring a double-layered structure. The outer layer facilitated the introduction of gas, while the inner layer allowed for the introduction of liquid. Just before reaching the catalyst packing area, the two phases were thoroughly mixed through the use of a PTFE filter or glass wool filter.

Procedure for hydrogenation of carbonyl compounds under continuous-flow conditions

In a manner analogous to our previous report,[1] heterogeneous catalyst (0.006 mmol as Pt) and Celite (0.5 g) were manually mixed in a glass vial through gentle shaking. The resulting mixed solid was then packed into a glass column, where the catalyst packing space measured 10 cm in length and had a 5 mm inner diameter for commercially available catalysts. The remaining space was filled with Celite. Pieces of glass wool were placed at both column ends, and the column was installed into the EYELA MCR-1000 column flow reactor (SynpleFlow™) with a double-layered column top unit for gas–liquid multi-phase reactions. To the column was fed 0.05 M of **1a** with decane (7–10 Mm, internal standard) in EtOAc using the Minatoconcept MCRP204 peristaltic pump unit (varied: 3.8–17 mL/h), and H_2 gas (fixed: 10 mL/min) was introduced simultaneously using the EYELA MFC-11GU mass flow controller in a downward flow direction. The flow rate was calculated by measuring the actually consumed volume of substrate solution in a fixed time. The flow reaction was conducted at room temperature to 80 °C, and the eluted solution was collected in a flask for a fixed period (every 30–120 min or 12 h (overnight running)). The continuous-flow reaction achieved a steady state after flowing under the same reaction conditions for more than 45

min. Following the continuous-flow of the substrate solution at a specific flow rate for a duration, the flow rate was subsequently adjusted to different values, and the resulting eluted solution was collected for analysis under the established steady state conditions.
Substrate solution includes internal standard (decane), and a collected fraction eluted from the flow reactor system was directly analyzed by GC-2030 (Shimadzu).
Isolation of products was conducted by simple evaporation of an eluted fraction and drying under vacuum. In this case, internal standard was not included in the solution. The structure of isolated compound was identified by comparison of ^{1}H and ^{13}C NMR spectra with commercially available samples or already reported data in literature.

Details of the investigation using heterogeneous catalysts in Table 1 is summarized below. Flow rate of **1a** solution was gradually increased in time course, and results were collected at each flow rate.

Definition of parameters:

Yield = conversion × selectivity.

MHSV (molar hourly space velocity (/h)) = flow rate of substrate solution (L/h) × substrate concentration (M)/Pt (mol) in a column

TOF (turnover frequency of catalyst (/h)) = MHSV (/h) × yield

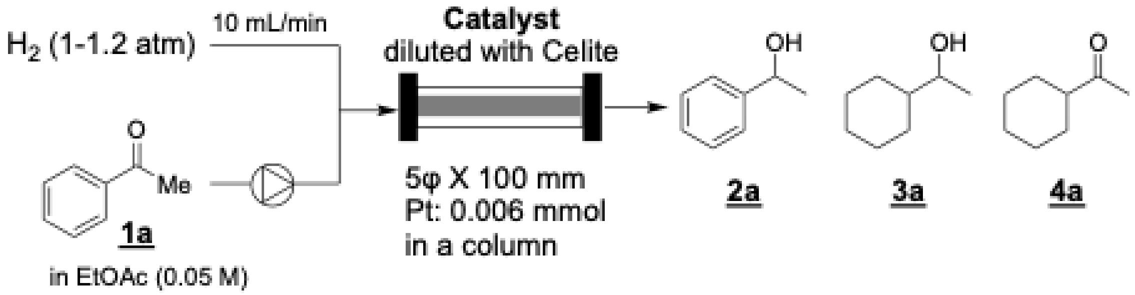

Representative results with different flow rates for each heterogeneous catalyst were summarized in tables below.

Table S1. Pt/C in a column

Flow rate (mL/h)	MHSV (/h)	**2a** (%)[a]	**3a** (%)[a]	**4a** (%)[a]	**1a** (%)[a]
4.0	33	16	2	10	72
7.6	63	7	1	6	83
9.4	78	4	0.4	3	92
13	125	3	0.3	2	95

[a] Determined by GC analysis with decane as internal standard

Table S2. Pt/SiO_2 in a column

Flow rate (mL/h)	MHSV (/h)	**2a** (%)[a]	**3a** (%)[a]	**4a** (%)[a]	**1a** (%)[a]
6.8	57	2	0	3	95
9.0	75	1	0	2	97
14	117	0.6	0	0.7	99

[a] Determined by GC analysis with decane as internal standard

Table S3. Pt/Al_2O_3 in a column

Flow rate (mL/h)	MHSV (/h)	**2a** (%)[a]	**3a** (%)[a]	**4a** (%)[a]	**1a** (%)[a]
5.0	42	36	5	13	47
9.0	75	17	1	9	73
13.2	110	10	1	5	85
16	133	5	0	3	92

[a] Determined by GC analysis with decane as internal standard

Table S4. Pt/DMPSi–Al_2O_3 in a column

Flow rate (mL/h)	MHSV (/h)	**2a** (%)[a]	**3a** (%)[a]	**4a** (%)[a]	**1a** (%)[a]
5.0	42	9	1	7	84
6.6	55	3	0	3	94
10.8	90	2	0	2	96
13	108	1	0	1	97

[a] Determined by GC analysis with decane as internal standard

Table S5. Pt–Au/DMPSi–Al_2O_3 in a column

Flow rate (mL/h)	MHSV (/h)	**2a** (%)[a]	**3a** (%)[a]	**4a** (%)[a]	**1a** (%)[a]
4.2	35	38	3	12	47
7.2	60	20	1	8	71
11	92	10	1	5	84

[a] Determined by GC analysis with decane as internal standard

Table S6. Pt–Ni/DMPSi–Al_2O_3 in a column

Flow rate (mL/h)	MHSV (/h)	**2a** (%)[a]	**3a** (%)[a]	**4a** (%)[a]	**1a** (%)[a]
5.4	45	32	2	0	66
9.2	77	14	1	0	85
14	117	7	0	0	93

[a] Determined by GC analysis with decane as internal standard

Table S7. Pt–Co/DMPSi–Al_2O_3 in a column

Flow rate (mL/h)	MHSV (/h)	**2a** (%)[a]	**3a** (%)[a]	**4a** (%)[a]	**1a** (%)[a]
4.6	38	81	2	0	17
13	108	34	1	0	65
17	142	16	0	0	84

[a] Determined by GC analysis with decane as internal standard

Table S8. Pt–Fe/DMPSi–Al_2O_3 (Fe/Pt = 0.62) in a column

Flow rate (mL/h)	MHSV (/h)	**2a** (%)[a]	**3a** (%)[a]	**4a** (%)[a]	**1a** (%)[a]
4.4	37	95	5	0	0
6.4	53	90	3	0	7
10.2	85	62	2	0	36
12.4	103	32	1	0	57
14.5	122	21	1	0	67

[a] Determined by GC analysis with decane as internal standard

Table S9. Pt–Fe/DMPSi–Al_2O_3 (Fe/Pt = 1.4) in a column

Flow rate (mL/h)	MHSV (/h)	**2a** (%)[a]	**3a** (%)[a]	**4a** (%)[a]	**1a** (%)[a]
3.8	32	97	2	0	1
6.4	53	71	1	0	28
9.6	80	46	0	0	54
12	100	32	0	0	68

[a] Determined by GC analysis with decane as internal standard

Table S10. Pt–Fe/DMPSi–Al_2O_3 (Fe/Pt = 2.3) in a column

Flow rate (mL/h)	MHSV (/h)	**2a** (%)[a]	**3a** (%)[a]	**4a** (%)[a]	**1a** (%)[a]
4.7	39	>99	0	0	0
6.6	55	90	0	0	10
8.4	70	68	0	0	32
12	100	45	0	0	55
15	125	33	0	9	67

[a] Determined by GC analysis with decane as internal standard

Table S11. Pt-Fe/DMPSi-Al_2O_3 (Fe/Pt=4.4) in a column

Flow rate (mL/h)	MHSV (/h)	**2a** (%)[a]	**3a** (%)[a]	**4a** (%)[a]	**1a** (%)[a]
4.2	35	74	0	0	26
6.8	57	25	0	0	75
10.2	85	10	0	0	90
14.2	118	5	0	0	95

[a] Determined by GC analysis with decane as internal standard

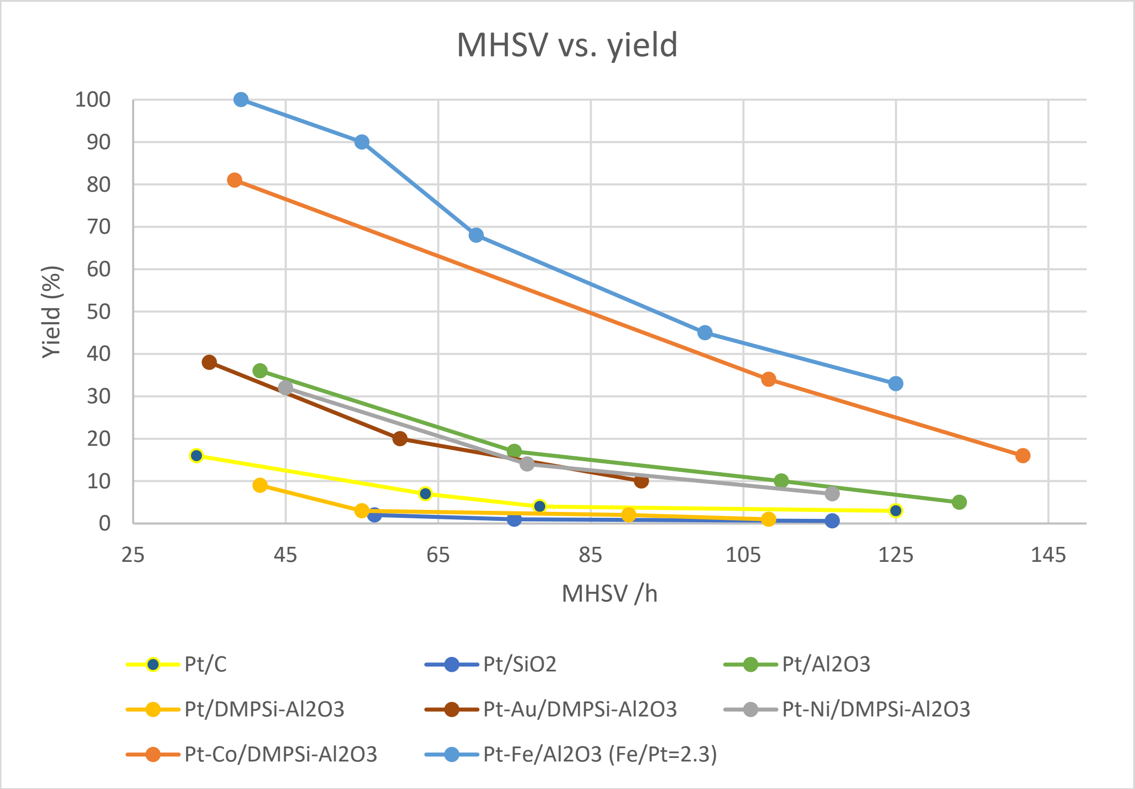

Figure S3. Comparison of catalysts (MHSV vs. yield)

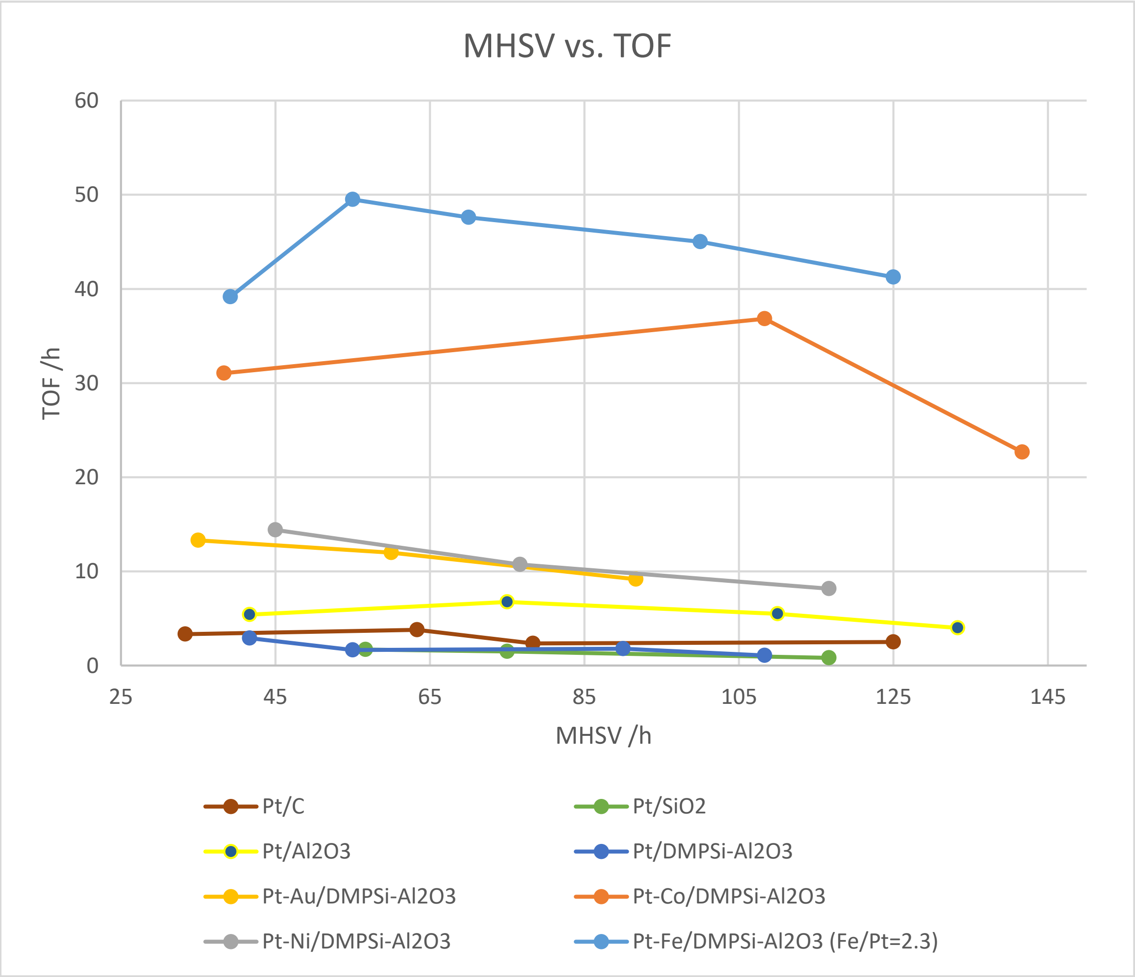

Figure S4. Comparison of catalysts (MHSV vs. TOF).

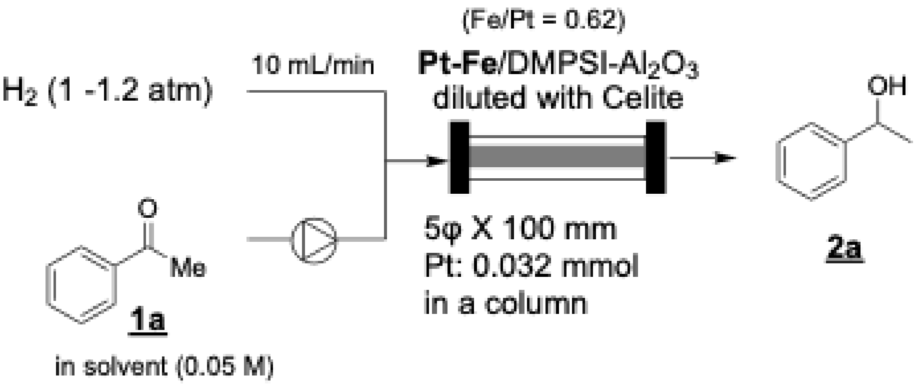

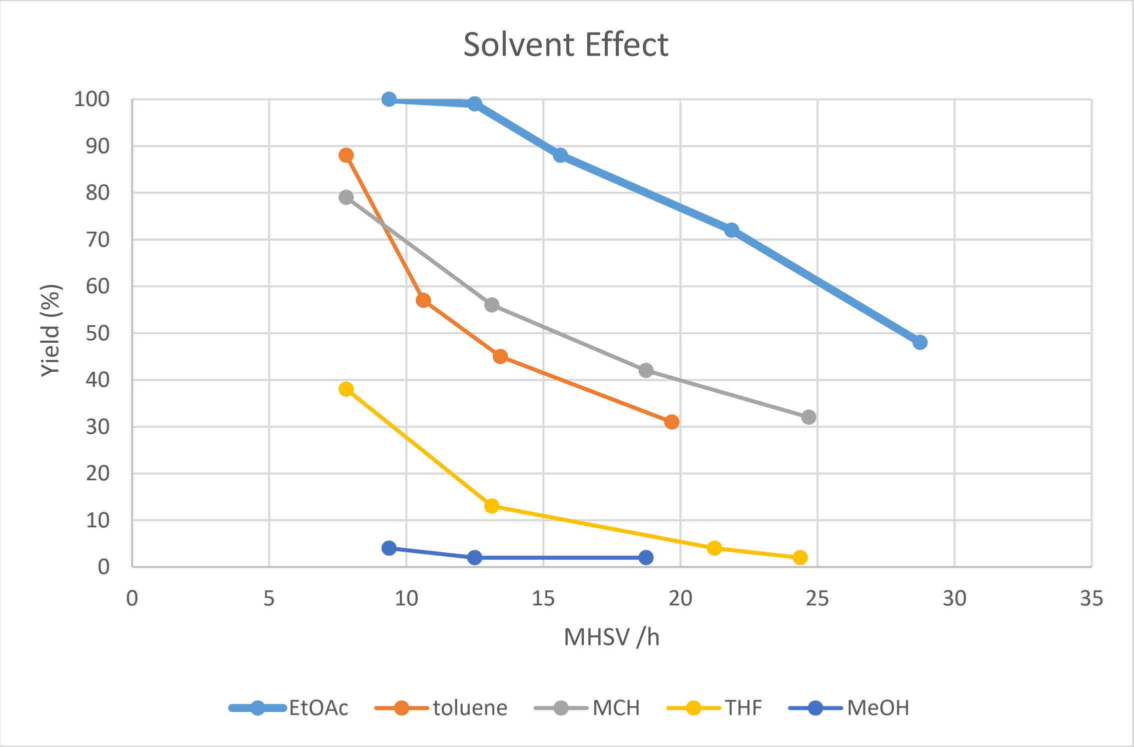

Figure S5. Comparison of solvents (MHSV vs. Yield)

Under sub-atmospheric hydrogen pressure conditions (Table 3, entry 1)

Heterogeneous catalyst: Pt–Fe/DMPSi–Al_2O_3 (Pt: 0.075 mmol/g Fe: 0.050 mmol/g, Fe/Pt = 0.67) 645 mg

Substrate solution: 0.05 M

H_2: 0.2–0.24 atm, N_2: 0.8–0.96 atm, total 20 mL/min

Flow rate (mL/h)	MHSV (/h)	**2a** (%)[a]	**1a** (%)[a]
3.8	3.9	>99	0
4.8	5.0	99	1
5.6	5.8	95	5
6.5	6.7	84	16
10.4	10.7	58	42

Detailed results of substrate scope varying MHSV or isolation of products

Hydrogenation of 1b

Heterogeneous catalyst: Pt–Fe/DMPSi-Al_2O_3 (Pt: 0.075 mmol/g Fe: 0.050 mmol/g, Fe/Pt = 0.67) 435 mg

Substrate solution: 0.040 M

H_2: 1–1.2 atm, 10 mL/min

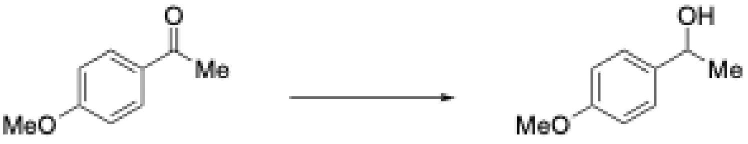

1b **2b**

Flow rate (mL/h)	MHSV (/h)	**2b** (%)[a]	**1b** (%)[a]
4.7	5.8	>99	0
7.2	8.8	91	9
11	13.5	70	30
13.3	16.3	52	48
16.6	20.4	38	61

[a] Determined by GC analysis with decane as internal standard

Hydrogenation of 1c

Heterogeneous catalyst: Pt-Fe/DMPSi-Al_2O_3 (Pt: 0.075 mmol/g Fe: 0.050 mmol/g, Fe/Pt = 0.67) 430 mg

Substrate solution: 0.045 M

H_2: 1–1.2 atm, 10 mL/min

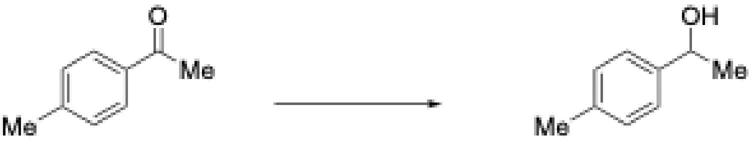

1c **2c**

Flow rate (mL/h)	MHSV (/h)	**2c** (%)[a]	**1c** (%)[a]
4.5	6.3	>99	0
7.0	9.8	94	6
11.7	16.3	79	21
13.4	18.7	69	31
15.4	21.5	56	44

[a] Determined by GC analysis with decane as internal standard

Hydrogenation of 1d

Heterogeneous catalyst: Pt-Fe/DMPSi-Al_2O_3 (Pt: 0.075 mmol/g Fe: 0.050 mmol/g, Fe/Pt = 0.67) 430 mg

Substrate solution: 0.035 M

H_2: 1-1.2 atm, 10 mL/min

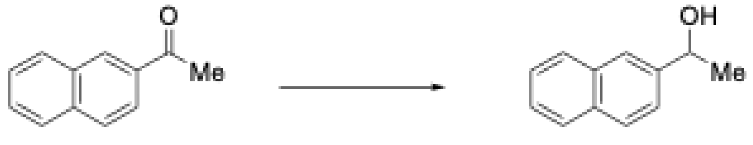

1d **2d**

At room temperature

Flow rate (mL/h)	MHSV (/h)	**2d** (%)[a]	**1d** (%)[a]
5.5	6.0	89	11
9.0	9.8	44	56
11.3	12.3	24	76
16.0	17.4	15	85
17.5	19.0	10	90

[a] Determined by GC analysis with decane as internal standard

At 50 °C

Flow rate (mL/h)	MHSV (/h)	**2d** (%)[a]	**1d** (%)[a]
5.4	5.9	>99	0
7.5	8.1	87	13
14.0	15.2	52	48
16.0	17.4	33	67
19.5	21.1	23	77

[a] Determined by GC analysis with decane as internal standard

Isolation of **2d**

Fixed flow rate: 3.8 mL/h at 50 °C

Fraction	Start time (h)	End time (h)	Yield **2d** (%)[a]
1	0	1.5	>99
2	1.5	3	>99
3	3	5	>99
4	5	8	>99
5	8	9	>99

[a] Isolated yield.

1-(2-Naphtyl)ethanol (**2d**) (identified by comparison with commercially available sample)

^{1}H NMR (400 MHz, $CDCl_3$) δ 7.81 (ddd, *J* = 8.8, 5.1, 1.9 Hz, 4H), 7.54 – 7.41 (m, 3H), 5.05 (q, *J* = 6.4 Hz, 1H), 1.97 (s, 1H), 1.57 (d, *J* = 6.5 Hz, 3H).

^{13}C NMR (101 MHz, $CDCl_3$) δ 143.21, 133.36, 132.96, 128.33, 127.95, 127.69, 126.16, 125.81, 123.84, 123.82, 70.55, 25.14.

Hydrogenation of 1e

Heterogeneous catalyst: Pt–Fe/DMPSi–Al_2O_3 (Pt: 0.075 mmol/g Fe: 0.050 mmol/g, Fe/Pt = 0.67) 430 mg

Substrate solution: 0.041 M

H_2: 1–1.2 atm, 10 mL/min

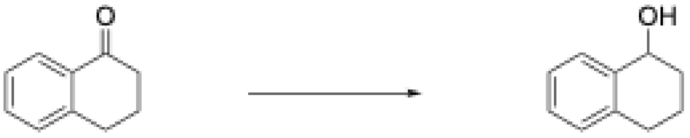

1e **2e**

Flow rate (mL/h)	MHSV (/h)	**2e** (%)[a]	**1e** (%)[a]
9.1	11.6	88	12
11.5	14.6	52	48
14.1	17.9	38	62
18.5	23.5	27	73

[a] Determined by GC analysis with decane as internal standard

Hydrogenation of 1f

Heterogeneous catalyst: Pt–Fe/DMPSi–Al_2O_3 (Pt: 0.075 mmol/g Fe: 0.050 mmol/g, Fe/Pt = 0.67) 430 mg

Substrate solution: 0.031 M

H_2: 1–1.2 atm, 10 mL/min

Fixed flow rate: 5.7 mL/h

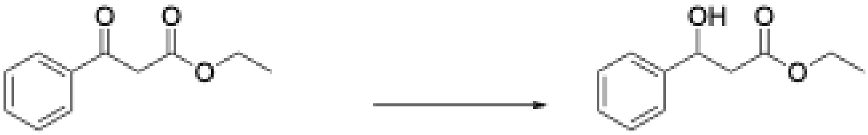

1f **2f**

Fraction	Start time (h)	End time (h)	Yield **2f** (%)[a]
1	0	1	>99
2	1	2	>99
3	2	3	>99
4	3	4	>99
5	4	5.5	>99
6	5.5	6.5	>99

[a] Isolated yield.

Ethyl 3-hydroxy-3-phenylpropanate (**2f**) (identified by comparison with commercially available sample)

^{1}H NMR (400 MHz, $CDCl_3$) δ 7.39 – 7.30 (m, 4H), 7.30 – 7.24 (m, 1H), 5.12 (dd, *J* = 8.7, 4.2 Hz, 1H), 4.17 (q, *J* = 7.1 Hz, 2H), 3.24 (s, 1H), 2.78 – 2.66 (m, 2H), 1.24 (t, *J* = 7.1 Hz, 3H).

^{13}C NMR (101 MHz, $CDCl_3$) δ 172.41, 142.53, 128.55, 127.80, 125.68, 70.35, 60.87, 43.35, 14.15.

Hydrogenation of 1g

Heterogeneous catalyst: Pt–Fe/DMPSi–Al_2O_3 (Pt: 0.014 mmol/g Fe: 0.020 mmol/g, Fe/Pt = 1.4) 428 mg

Substrate solution: 0.041 M

H_2: 1–1.2 atm, 10 mL/min

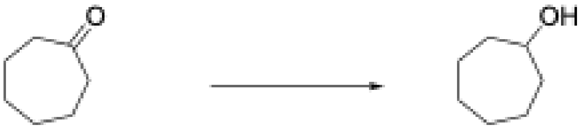

1g **2g**

Flow rate (mL/h)	MHSV (/h)	**2g** (%)[a]	**1g** (%)[a]
5.9	40.2	95	5
8.0	54.5	79	21
10.2	69.5	65	35
14.0	95.3	47	53

[a] Determined by GC analysis with decane as internal standard

Hydrogenation of 1h

Heterogeneous catalyst: Pt–Fe/DMPSi–Al_2O_3 (Pt: 0.075 mmol/g Fe: 0.050 mmol/g, Fe/Pt = 0.67) 430 mg

Substrate solution: 0.062 M

H_2: 1–1.2 atm, 10 mL/min

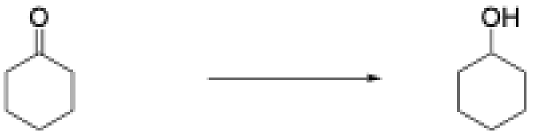

1h **2h**

Flow rate (mL/h)	MHSV (/h)	**2h** (%)[a]	**1h** (%)[a]
8.8	16.9	>99	0
11.0	21.1	>99	0
14.5	27.9	>99	0
18.2	35.0	>99	0

[a] Determined by GC analysis with decane as internal standard

Hydrogenation of 1i

Heterogeneous catalyst: Pt–Fe/DMPSi–Al_2O_3 (Pt: 0.075 mmol/g Fe: 0.050 mmol/g, Fe/Pt = 0.67) 430 mg

Substrate solution: 0.072 M

H_2: 1–1.2 atm, 10 mL/min

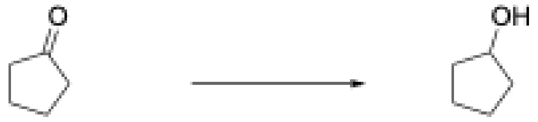

1i **2i**

Flow rate (mL/h)	MHSV (/h)	**2i** (%)[a]	**1i** (%)[a]
7.3	16.3	98	2
11.4	25.5	84	16
13.8	30.8	67	33
19.1	42.6	47	53

[a] Determined by GC analysis with decane as internal standard

Under sub-atmospheric hydrogen pressure conditions (Table 3, entries 2, 3)

Heterogeneous catalyst: Pt–Fe/DMPSi–Al_2O_3 (Pt: 0.075 mmol/g Fe: 0.050 mmol/g, Fe/Pt = 0.67) 430 mg

Substrate solution: 0.072 M

H_2: 0.2–0.24 atm, N_2: 0.8–0.96 atm, total 20 mL/min

Flow rate (mL/h)	MHSV (/h)	**2i** (%)[a]	**1i** (%)[a]
6.9	15.4	97	3
8.0	17.9	96	4

Heterogeneous catalyst: Pt–Fe/DMPSi–Al_2O_3 (Pt: 0.075 mmol/g Fe: 0.050 mmol/g, Fe/Pt = 0.67) 430 mg

Substrate solution: 0.072 M

H_2: 0.1–0.12 atm, N_2: 0.9–1.08 atm, total 20 mL/min

Flow rate (mL/h)	MHSV (/h)	**2i** (%)[a]	**1i** (%)[a]
6.3	14.1	84	16

Hydrogenation of 1j

Heterogeneous catalyst: Pt–Fe/DMPSi–Al_2O_3 (Pt: 0.075 mmol/g Fe: 0.050 mmol/g, Fe/Pt = 0.67) 430 mg

Substrate solution: 0.047 M

H_2: 1–1.2 atm, 10 mL/min

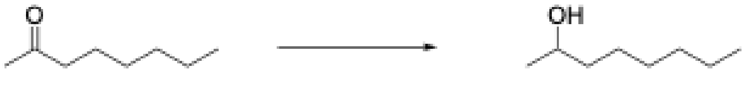

1j 2j

Flow rate (mL/h)	MHSV (/h)	**2j** (%)[a]	**1j** (%)[a]
4.7	6.8	>99	0
8.8	12.8	91	9
13.7	20.0	89	11
15.8	23.0	71	29
19.5	28.4	51	49

[a] Determined by GC analysis with decane as internal standard

Hydrogenation of 1k

Heterogeneous catalyst: Pt–Fe/DMPSi–Al_2O_3 (Pt: 0.025 mmol/g Fe: 0.058 mmol/g, Fe/Pt = 2.3) 240 mg

Substrate solution: 0.016 M

H_2: 1–1.2 atm, 10 mL/min

Fixed flow rate: 4.3 mL/h, MHSV: 12 /h

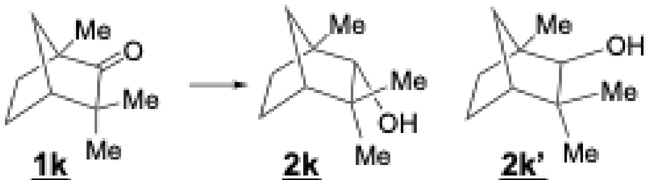

1k 2k 2k'

Temperature ºC	Yield of **2k** (%)	Yield of **2k'**(%)
50	3	14
60	5	18
70	19	48
80	25	49
90	21	41

[a] Determined by GC analysis with decane as internal standard

Hydrogenation of 1l

Heterogeneous catalyst: Pt–Fe/DMPSi–Al_2O_3 (Pt: 0.025 mmol/g Fe: 0.058 mmol/g, Fe/Pt = 2.3) 240 mg

Substrate solution: 0.01 M

H_2: 1–1.2 atm, 10 mL/min

Fixed flow rate: 4.7 mL/h, MHSV: 8 /h

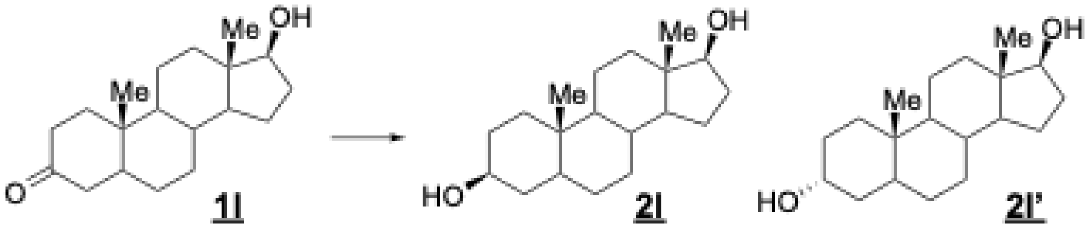

Fraction	Start time (h)	End time (h)	Yield **2l** (%)[a]	Yield **2l'** (%)[a]
1	0	1.5	77	23
2	1.5	2.5	75	25
3	2.5	4.5	74	26
4	4.5	6	74	26
5	6	8	74	26
6	8	9	74	26

[a] Obtained as mixture of isomers. Ratio of isomers was determined by ^{1}H NMR analysis[2].

Major isomer (2l) ^{1}H NMR (400 MHz, $CDCl_3$) δ 3.56 – 3.65 (m, 2H), 2.00 – 2.10 (m, 1H), 1.64 – 1.85 (m, 4H), 1.20 – 1.50 (m, 12H), 0.85 – 1.20 (m, 6H), 0.82 (3H, s), 0.73 (3H, s), δ 0.63 (ddd, J = 12.3, 10.4, 4.1 Hz, 1H).[2]

Hydrogenation of 1m

Heterogeneous catalyst: Pt–Fe/DMPSi–Al_2O_3 (Pt: 0.014 mmol/g Fe: 0.020 mmol/g, Fe/Pt = 0.67) 428 mg

Substrate solution: 0.057 M

H_2: 1–1.2 atm, 10 mL/min

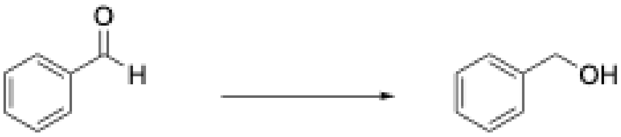

1m **2m**

Flow rate (mL/h)	MHSV (/h)	**2m** (%)[a]	**1m** (%)[a]
7.1	67.5	98	2
9.0	85.6	98	2
10.0	95.1	99	1
13.6	129.4	99	1
18.9	179.8	>99	0

[a] Determined by GC analysis with decane as internal standard

Hydrogenation of 1n

Heterogeneous catalyst: Pt–Fe/DMPSi–Al_2O_3 (Pt: 0.014 mmol/g Fe: 0.020 mmol/g, Fe/Pt = 0.67) 428 mg

Substrate solution: 0.05 M

H_2: 1–1.2 atm, 10 mL/min

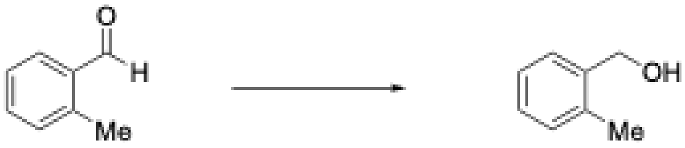

1n **2n**

Flow rate (mL/h)	MHSV (/h)	**2n** (%)[a]	**1n** (%)[a]
6.0	50.1	98	2
8.7	72.6	>99	0
12.8	106.8	91	9
15.0	125.2	83	17

[a] Determined by GC analysis with decane as internal standard

Hydrogenation of 1o

Heterogeneous catalyst: Pt–Fe/DMPSi–Al_2O_3 (Pt: 0.014 mmol/g Fe: 0.020 mmol/g, Fe/Pt = 0.67) 428 mg

Substrate solution: 0.05 M

H_2: 1–1.2 atm, 10 mL/min

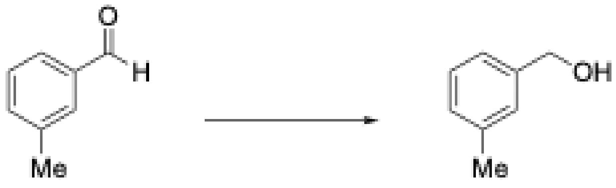

1o **2o**

Flow rate (mL/h)	MHSV (/h)	**2o** (%)[a]	**1o** (%)[a]
6.0	50.1	>99	0
8.4	70.1	>99	0
12.8	106.8	98	2
16.5	137.7	93	7
19.7	164.4	87	13

[a] Determined by GC analysis with decane as internal standard

Hydrogenation of 1p

Heterogeneous catalyst: Pt–Fe/DMPSi–Al_2O_3 (Pt: 0.014 mmol/g Fe: 0.020 mmol/g, Fe/Pt = 0.67) 428 mg

Substrate solution: 0.05 M

H_2: 1–1.2 atm, 10 mL/min

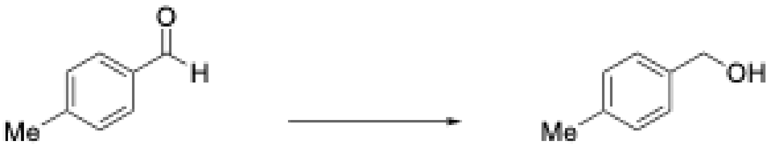

1p **2p**

Flow rate (mL/h)	MHSV (/h)	**2p** (%)[a]	**1p** (%)[a]
5.7	47.6	>99	0
9.0	75.1	>99	0
12.5	104.3	95	5
16.4	136.8	86	14
20.9	174.4	74	26

[a] Determined by GC analysis with decane as internal standard

Hydrogenation of 1q

Heterogeneous catalyst: Pt–Fe/DMPSi–Al_2O_3 (Pt: 0.014 mmol/g Fe: 0.020 mmol/g, Fe/Pt = 0.67) 428 mg

Substrate solution: 0.03 M

H_2: 1–1.2 atm, 10 mL/min

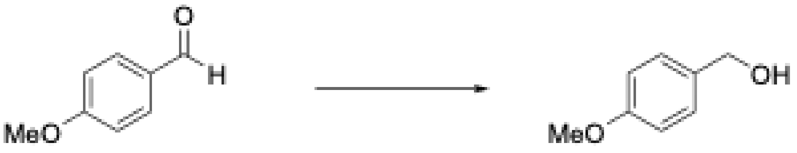

1q **2q**

Flow rate (mL/h)	MHSV (/h)	**2q** (%)[a]	**1q** (%)[a]
5.4	27.0	98	2
7.8	39.1	>99	0
9.9	49.6	>99	0
13.8	69.1	>99	0
17.7	88.6	>99	0

[a] Determined by GC analysis with decane as internal standard

Hydrogenation of 1r

Heterogeneous catalyst: Pt–Fe/DMPSi–Al_2O_3 (Pt: 0.014 mmol/g Fe: 0.020 mmol/g, Fe/Pt = 0.67) 428 mg

Substrate solution: 0.043 M

H_2: 1–1.2 atm, 10 mL/min

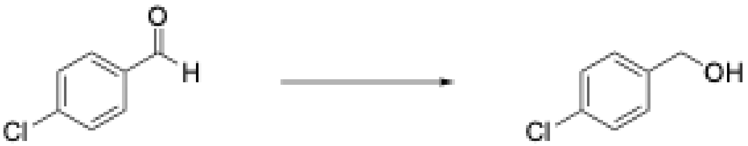

1r **2r**

Flow rate (mL/h)	MHSV (/h)	**2r** (%)[a]	**1r** (%)[a]
5.8	41.6	94	0
10.2	73.2	96	0
13.3	95.4	97	0
18.2	130.6	98	0

[a] Determined by GC analysis with decane as internal standard

Hydrogenation of 1s

Heterogeneous catalyst: Pt–Fe/DMPSi–Al_2O_3 (Pt: 0.075 mmol/g Fe: 0.050 mmol/g, Fe/Pt = 0.67) 430 mg

Substrate solution: 0.038 M

H_2: 1–1.2 atm, 10 mL/min

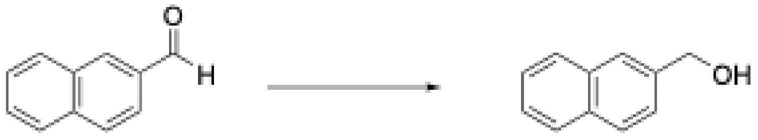

1s **2s**

Flow rate (mL/h)	MHSV (/h)	**2s** (%)[a]	**1s** (%)[a]
5.1	6.0	>99	0
7.6	9.0	>99	0
12.9	15.2	55	45
14.0	16.5	36	64

[a] Determined by GC analysis with decane as internal standard

Isolation of **2s**

Fixed flow rate: 6.8 mL/h at room temperature

Fraction	Start time (h)	End time (h)	Yield **2t** (%)[a]
1	0	0.83	>99
2	0.83	3.1	>99
3	3.1	4.1	98
4	4.1	5.4	>99
5	5.4	6.5	97
6	6.5	7.5	>99

[a] Isolated yield.

Naphthalen-2-ylmethanol (**2s**) (identified by comparison with commercially available sample)

^{1}H NMR (400 MHz, $CDCl_3$) δ 7.81 (ddd, *J* = 10.6, 6.0, 2.5 Hz, 4H), 7.58 – 7.39 (m, 3H), 4.84 (d, *J* = 5.4 Hz, 2H), 1.81 (t, *J* = 5.8 Hz, 1H).

^{13}C NMR (101 MHz, $CDCl_3$) δ 138.33, 133.41, 132.98, 128.36, 127.90, 127.73, 126.20, 125.92, 125.46, 125.17, 65.51.

Hydrogenation of 1t

Heterogeneous catalyst: Pt–Fe/DMPSi–Al_2O_3 (Pt: 0.075 mmol/g Fe: 0.050 mmol/g, Fe/Pt = 0.67) 430 mg

Substrate solution: 0.05 M

H_2: 1–1.2 atm, 10 mL/min

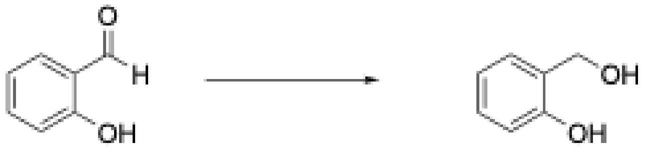

1t **2t**

Flow rate (mL/h)	MHSV (/h)	**2t** (%)[a]	**1t** (%)[a]
5.2	8.1	>99	0
8.7	13.5	>99	0
12.2	18.9	86	14
16.6	25.7	67	33
18.0	27.9	55	45

[a] Determined by GC analysis with decane as internal standard

Isolation of **2t**

Fixed flow rate: 6 mL/h at room temperature

Fraction	Start time (h)	End time (h)	Yield **2t** (%)[a]
1	0	0.67	>99
2	0.67	1.67	>99
3	1.67	2.5	>99
4	2.5	3.75	>99
5	3.75	5.5	>99
6	5.5	6.5	>99

[a] Isolated yield.

2-(Hydroxymethyl)phenol (**2t**) (identified by comparison with commercially available sample)

^{1}H NMR (400 MHz, $CDCl_3$) δ 7.3 (br 1H), 7.19 (td, J = 7.7, 1.7 Hz, 1H), 7.02 (dd, J = 7.5, 1.7 Hz, 1H), 6.92 – 6.76 (m, 2H), 4.83 (s, 2H), 2.42 (br, 1H).

^{13}C NMR (101 MHz, $CDCl_3$) δ 156.02, 129.52, 127.88, 124.72, 120.13, 116.53, 64.58.

Hydrogenation of 1u

Heterogeneous catalyst: Pt–Fe/DMPSi–Al_2O_3 (Pt: 0.075 mmol/g Fe: 0.050 mmol/g, Fe/Pt = 0.67) 430 mg

Substrate solution: 0.057 M

H_2: 1–1.2 atm, 10 mL/min

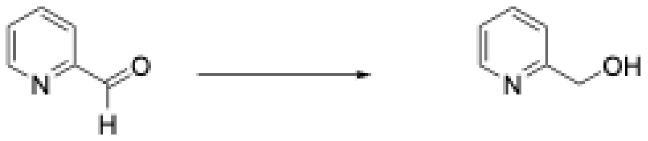

1u **2u**

Flow rate (mL/h)	MHSV (/h)	**2u** (%)[a]	**1u** (%)[a]
5.3	9.4	91	9
6.9	12.2	22	78
11.0	19.4	10	90
14.0	24.7	4	96

[a] Determined by GC analysis with decane as internal standard

Hydrogenation of 1v

Heterogeneous catalyst: Pt–Fe/DMPSi–Al_2O_3 (Pt: 0.075 mmol/g Fe: 0.050 mmol/g, Fe/Pt = 0.67) 430 mg

Substrate solution: 0.053 M

H_2: 1–1.2 atm, 10 mL/min

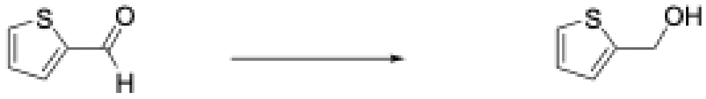

1v **2v**

Flow rate (mL/h)	MHSV (/h)	**2v** (%)[a]	**1v** (%)[a]
5.2	8.5	61	39
7.5	12.3	29	71
12.4	20.4	18	82
15.6	25.6	13	87
17.8	29.3	11	89

[a] Determined by GC analysis with decane as internal standard

Hydrogenation of 1w

Heterogeneous catalyst: Pt–Fe/DMPSi–Al_2O_3 (Pt: 0.075 mmol/g Fe: 0.050 mmol/g, Fe/Pt = 0.67) 430 mg

Substrate solution: 0.047 M

H_2: 1–1.2 atm, 10 mL/min

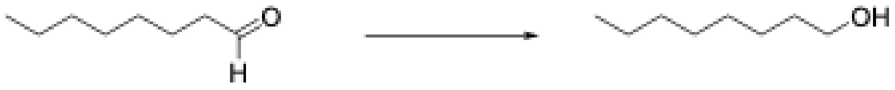

1w **2w**

Flow rate (mL/h)	MHSV (/h)	**2w** (%)[a]	**1w** (%)[a]
8.2	12.0	98	2
11.3	16.5	97	3
15.0	21.9	96	4
16.7	24.3	97	3

[a] Determined by GC analysis with decane as internal standard

Under sub-atmospheric hydrogen pressure conditions (Table 3, entry 5)

Heterogeneous catalyst: Pt–Fe/DMPSi–Al_2O_3 (Pt: 0.061 mmol/g Fe: 0.038 mmol/g, Fe/Pt = 0.62) 100 mg

Substrate solution: 0.032 M

H_2: 0.2–0.24 atm, N_2: 0.8–0.96 atm, total 10 mL/min

Flow rate (mL/h)	MHSV (/h)	**2w** (%)[a]	**1w** (%)[a]
4.0	21.0	95	0
5.9	31.0	90	9
8.8	46.1	75	25
12.0	63.0	46	54

Hydrogenation of 1x

Heterogeneous catalyst: Pt–Fe/DMPSi–Al_2O_3 (Pt: 0.075 mmol/g Fe: 0.050 mmol/g, Fe/Pt = 0.67) 430 mg

Substrate solution: 0.033 M

H_2: 1–1.2 atm, 10 mL/min

Fixed flow rate: 10.4 mL/h at room temperature

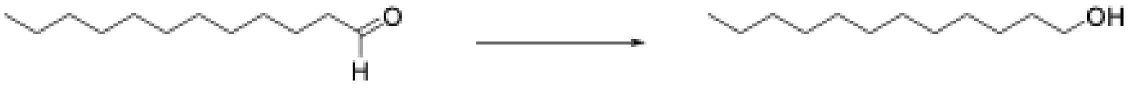

1x **2x**

Fraction	Start time (h)	End time (h)	Yield **2x** (%)[a]
1	0	0.75	>99
2	0.75	1.75	>99
3	1.75	2.75	>99
4	2.75	3.75	>99
5	3.75	5.25	>99
6	5.25	6.75	>99
7	6.75	7.75	>99
8	7.75	9	>99

[a] Isolated yield.

1-Dodecanol (**2x**) (identified by comparison with commercially available sample)

^{1}H NMR (400 MHz, $CDCl_3$) δ 3.61 (t, *J* = 6.6 Hz, 2H), 1.53 (dt, *J* = 8.1, 6.4 Hz, 2H), 1.37 – 1.15 (m, 19H), 0.91 – 0.81 (m, 3H).

^{13}C NMR (101 MHz, $CDCl_3$) δ 63.08, 32.82, 31.91, 29.66, 29.63, 29.61, 29.59, 29.44, 29.34, 25.75, 22.68, 14.09.

Hydrogenation of 1y

Heterogeneous catalyst: Pt–Fe/DMPSi–Al_2O_3 (Pt: 0.036 mmol/g Fe: 0.044 mmol/g, Fe/Pt = 1.2) 167 mg

Substrate solution: 0.03 M

H_2: 1–1.2 atm, 10 mL/min

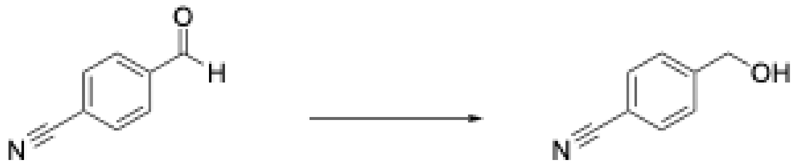

1y **2y**

Flow rate (mL/h)	MHSV (/h)	**2y** (%)[a]	**1y** (%)[a]
7.0	34.9	95	0
14.0	70.0	81	19
18.0	89.8	56	44

[a] Determined by GC analysis with decane as internal standard

Comparison of heterogeneous catalysts for hydrogenation of carbonyl compounds

Table S12. Comparison of heterogeneous catalysts for hydrogenation of carbonyl compounds

Catalyst	substrate	Temperature	H_2 pressure	Yield	Conversion	Selectivity	Reference
Pt/Reduced graphene oxide	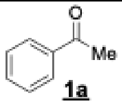 1a	40 ºC	40 atm	86%	>99%	86%	Ref. 3
ARP-Pt	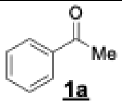 1a	40 ºC	1 atm	52%	>99%	52%	Ref. 4
Ru/FCN	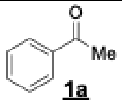 1a	40 ºC	1 atm	61%	79%	77%	Ref. 5
Ru-Pt/PVP	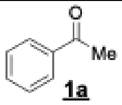 1a	30 ºC	200 KPa	71%	99%	72%	Ref. 6
Plated milling vessel	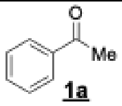 1a	rt	5 bar	51%	–	–	Ref. 7
**Pt-Fe/DMPSi-Al_2O_3**	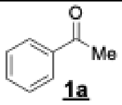 1a	**rt**	**1 atm (101 KPa)**	**>99%**	**>99%**	**>99%**	**This work**
**Pt-Fe/DMPSi-Al_2O_3**	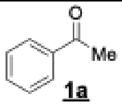 1a	**rt**	**0.24 atm (24 KPa)**	**93%**	**93%**	**>99%**	**This work**
Ru-Pt/PVP	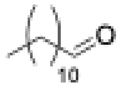	30 ºC	100 KPa	52%	-	-	Ref. 6
ARP-Pt	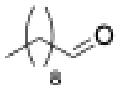	40 ºC	1 atm	17%	43%	40%	Ref. 4
Plated milling vessel	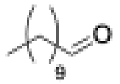	rt	5 bar	43%	-	-	Ref. 7
**Pt-Fe/DMPSi-Al_2O_3**	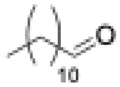	**rt**	**1 atm (101 kPa)**	**>99%**	**>99%**	**>99%**	**This work**
**Pt-Fe/DMPSi-Al_2O_3**	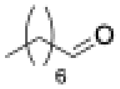	**rt**	**0.24 atm (24 KPa)**	**97%**	**97%**	**>99%**	**This work**

^{1}H and ^{13}C NMR spectra of isolated compounds

1-(2-Naphtyl)ethanol (2d)

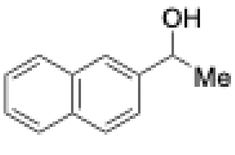

2d

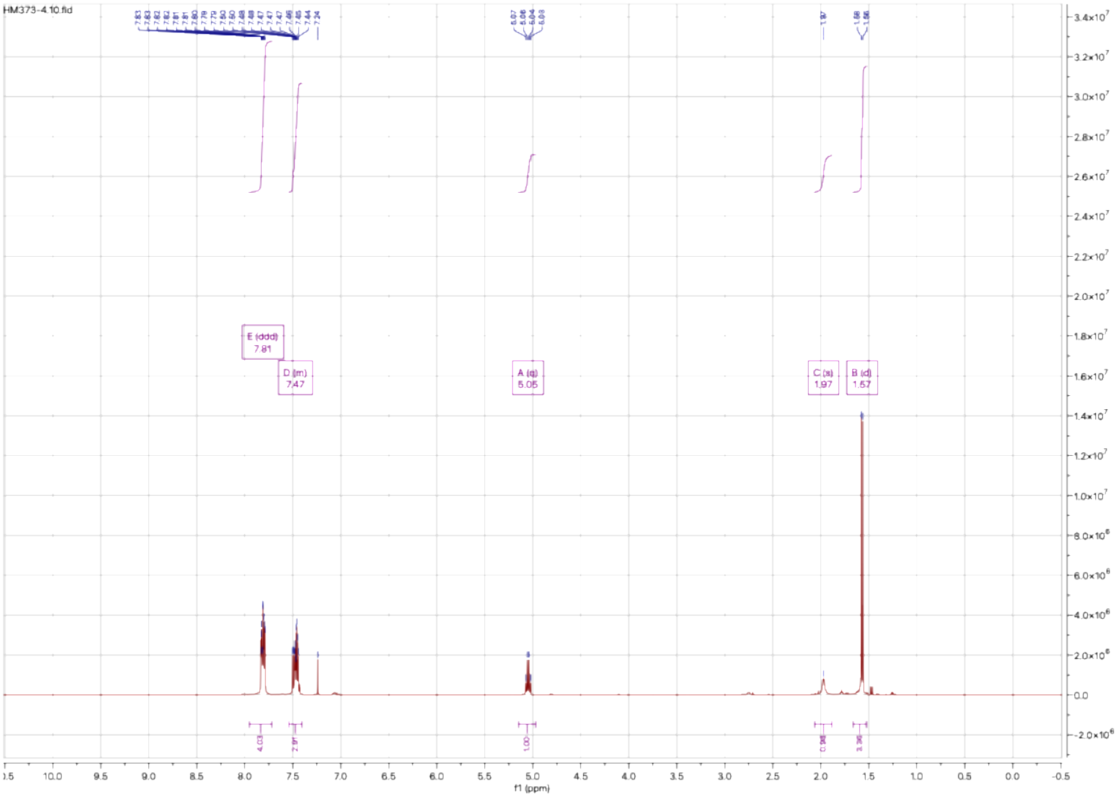

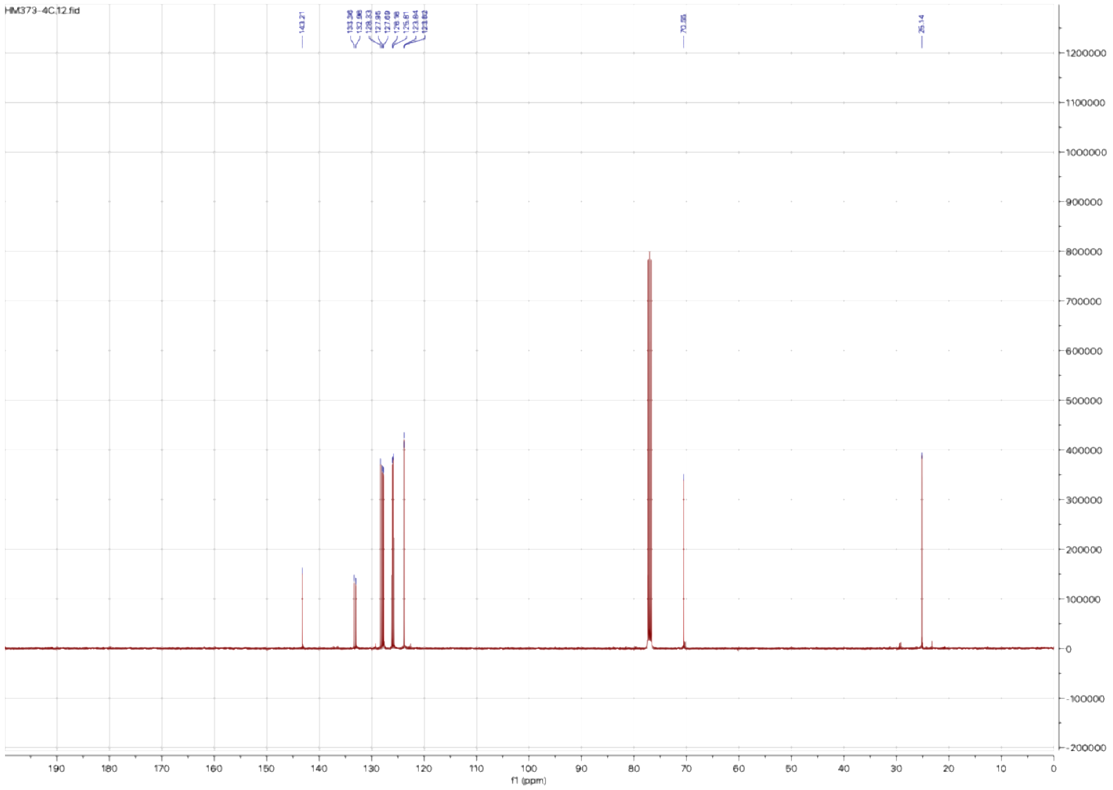

Ethyl 3-hydroxy-3-phenylpropanate (2f)

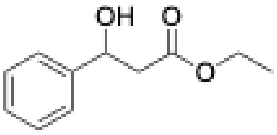

2f

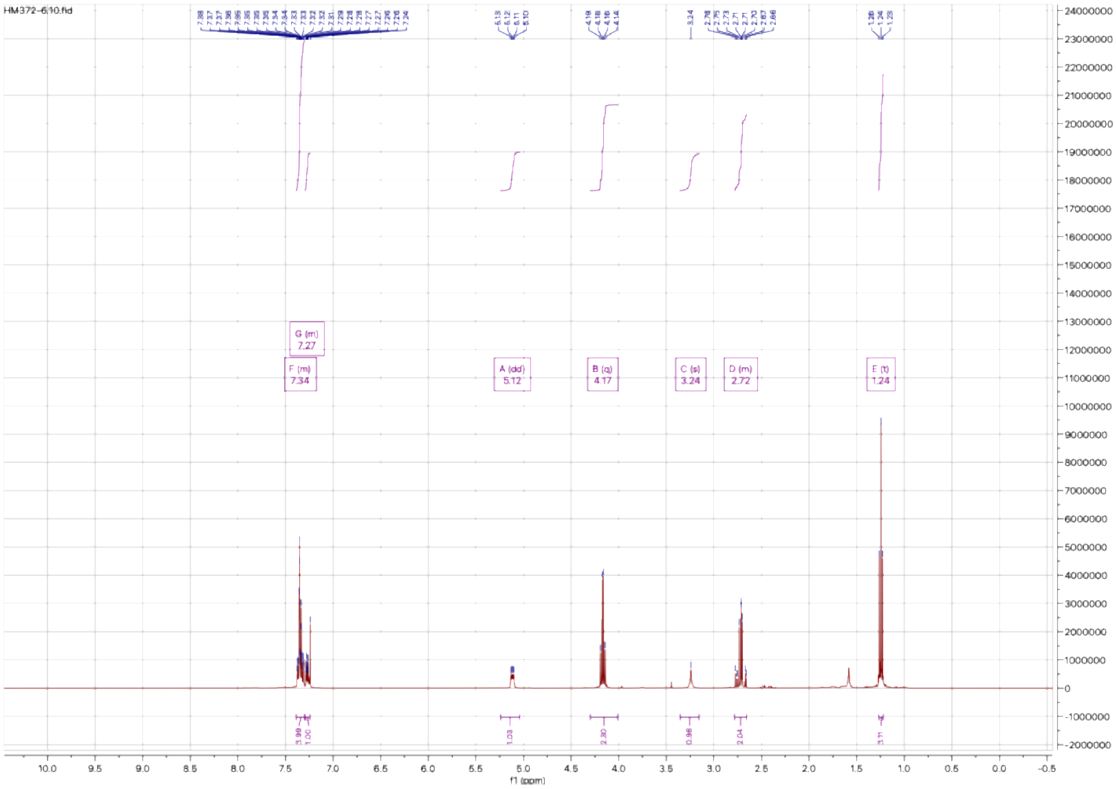

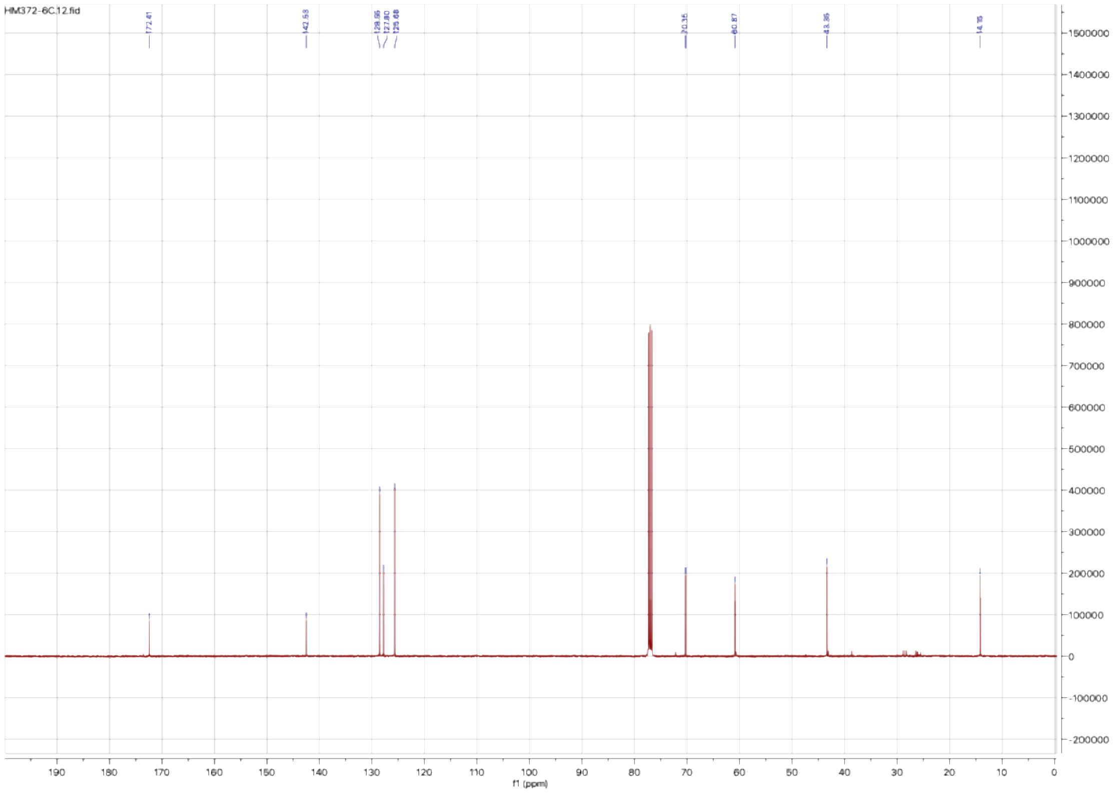

Naphthalen-2-ylmethanol (2s)

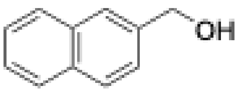

2s

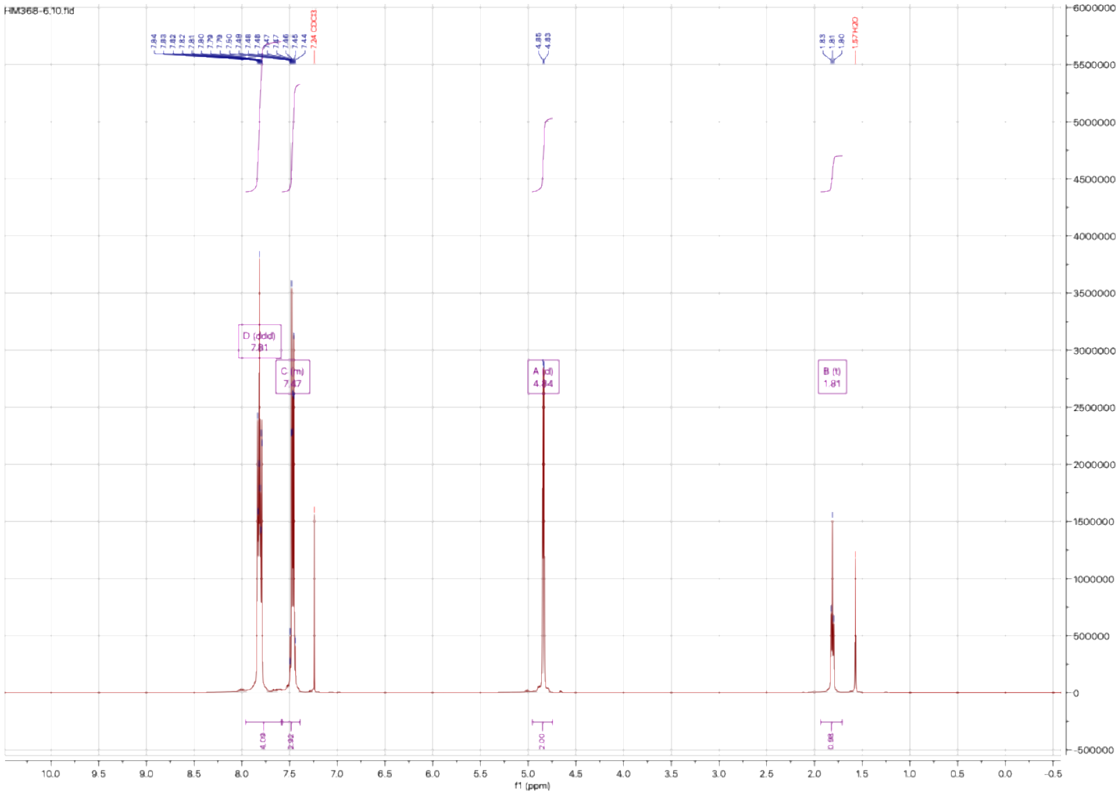

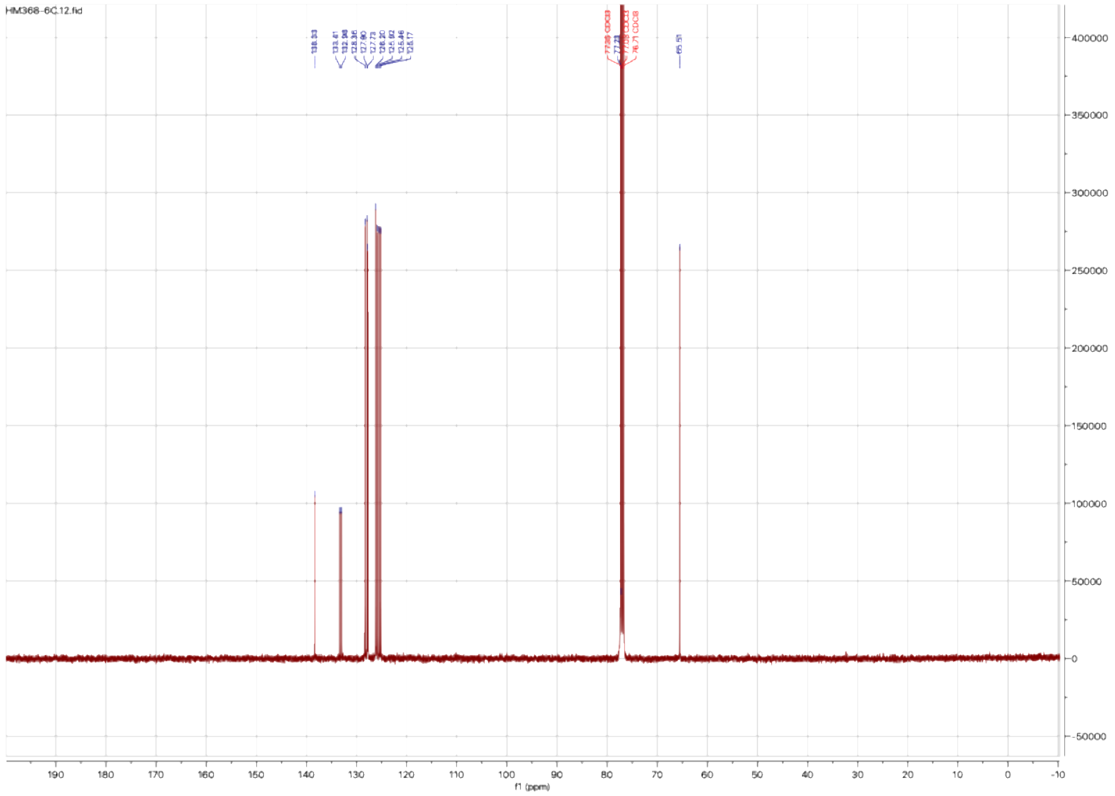

2-(Hydroxymethyl)phenol (2t)

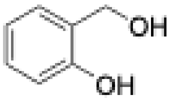

2t

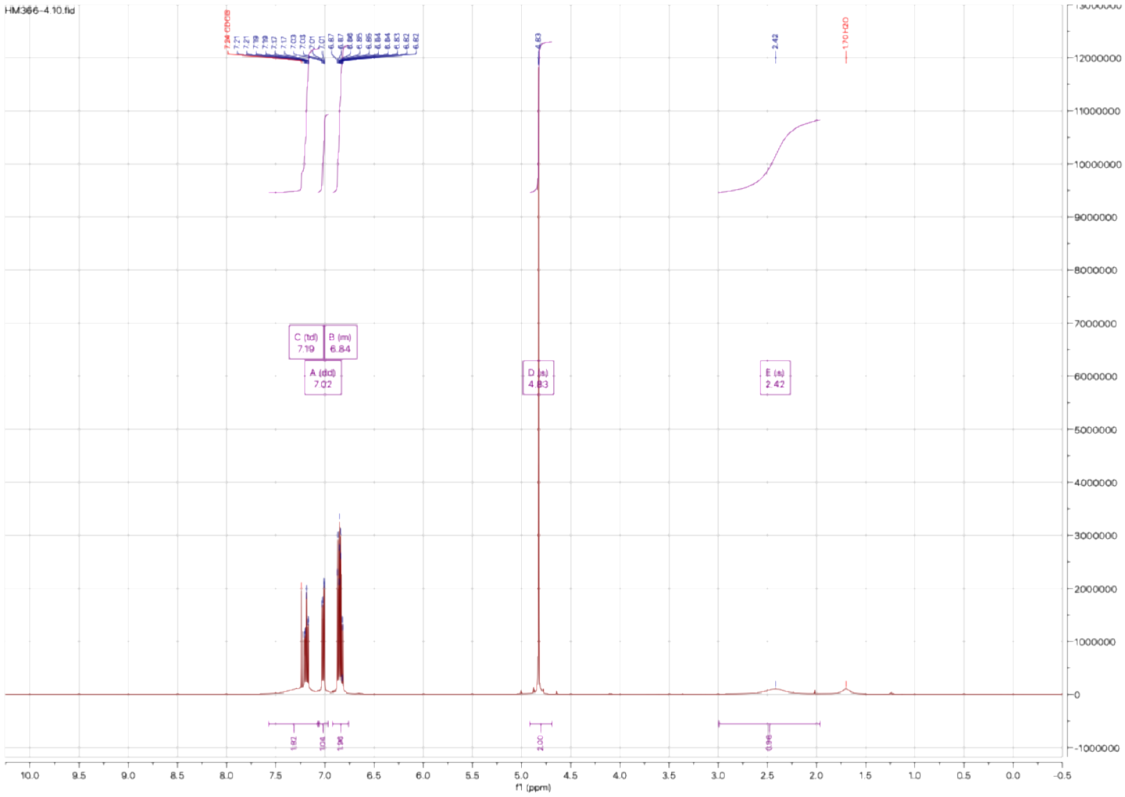

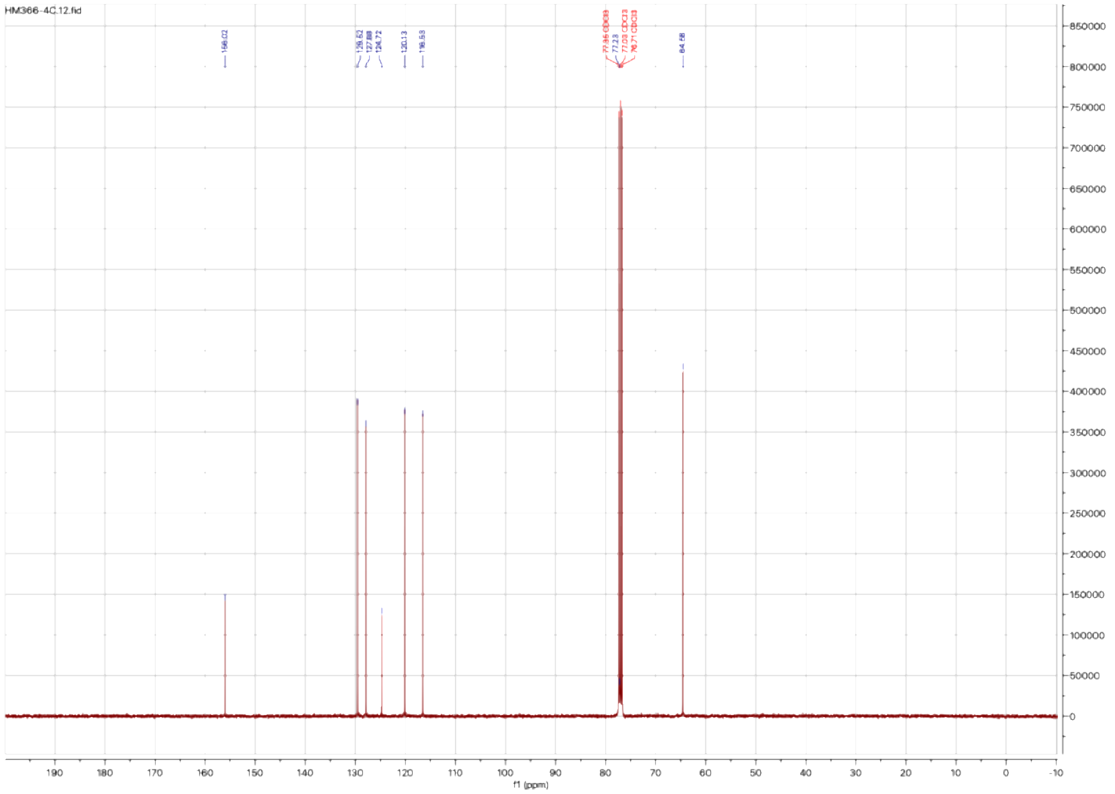

1-Dodecanol (2x)

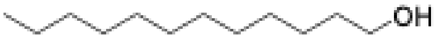

2x

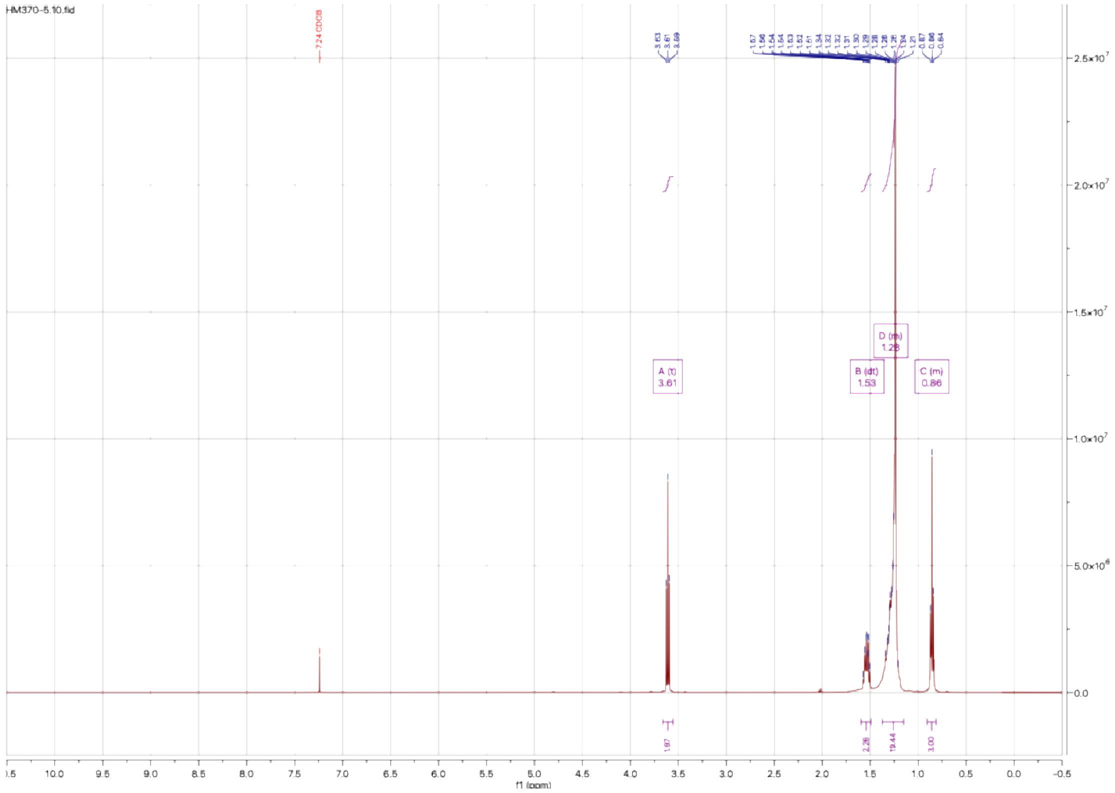

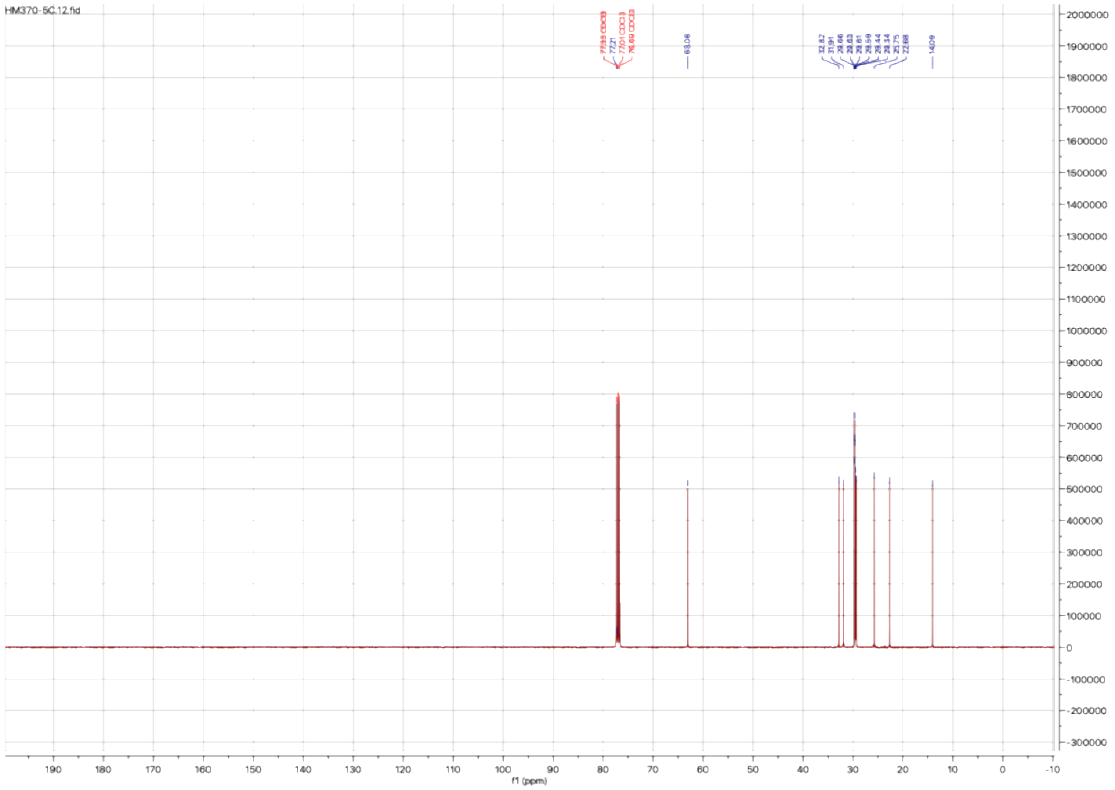

2l + 2l' [2]

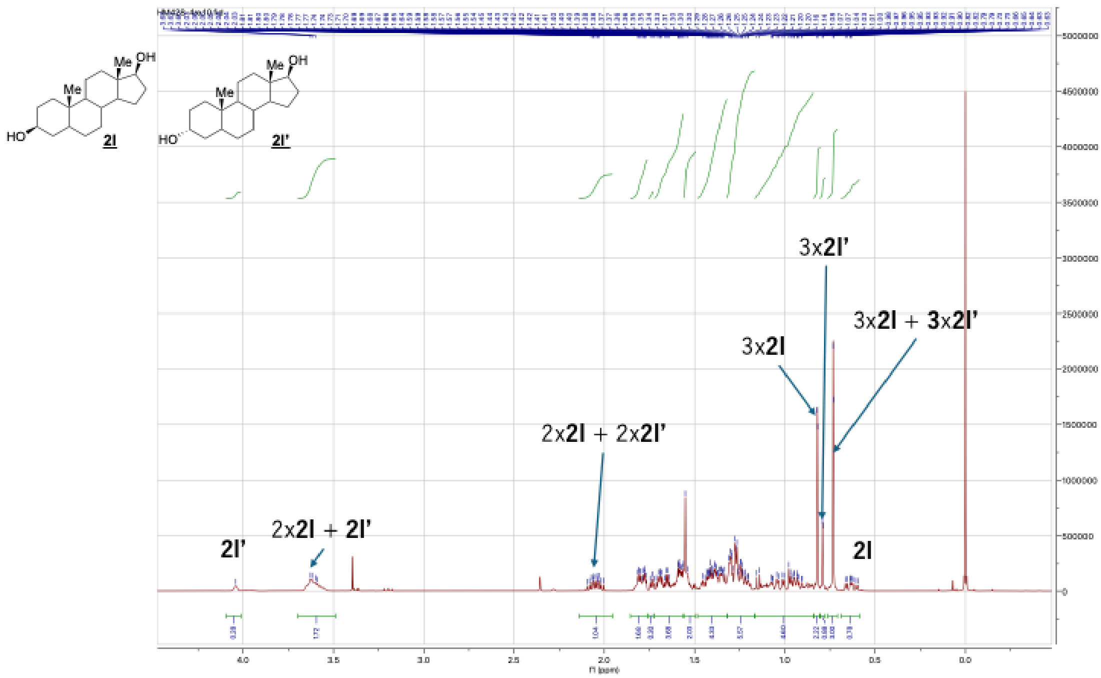

^{1}H NMR spectrum of stanolone (**1l**) (substrate)

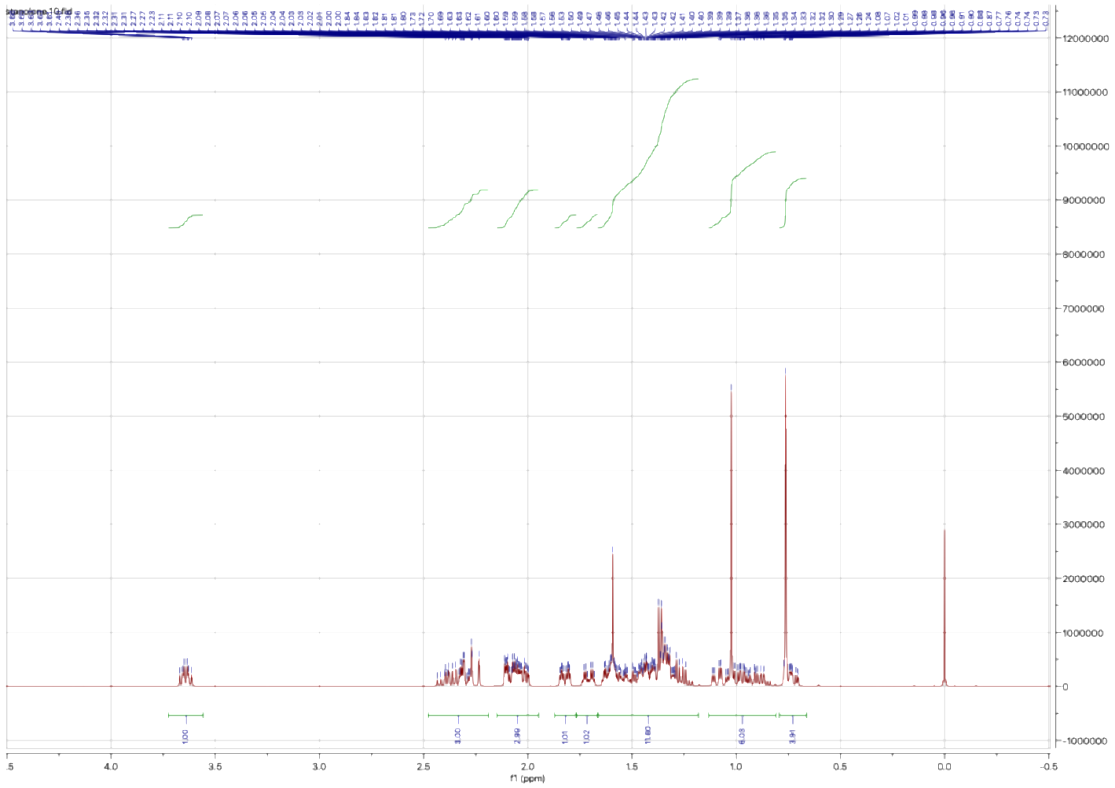

References

1) Miyamura, H.; Sharma, A.; Takata, M.; Kajiyama, R.; Kobayashi, S.; Kon, Y. Selective Hydrogenation of Quinizarins to Leuco-quinizarins and Their Direct Derivatization Using Flow-Batch-Separator Unified Reactors under Continuous-Flow Conditions. *ACS Catalysis* **2024**, *14*, 10317-10323.

2) Kamijo, S.; Tao, K.; Takao, G.; Tonoda, H.; Murafuji, T. Photoinduced Oxidation of Secondary Alcohols Using 4-Benzoylpyridine as an Oxidant. *Organic Letters* **2015**, *17*, 3326-3329.

3) Tan, J.; Cui, J.; Cui, X.; Deng, T.; Li, X.; Zhu, Y.; Li, Y. Graphene-Modified Ru Nanocatalyst for Low-Temperature Hydrogenation of Carbonyl Groups. *ACS Catalysis* **2015**, *5*, 7379-7384.

4) Osako, T.; Torii, K.; Hirata, S.; Uozumi, Y. Chemoselective Continuous-Flow Hydrogenation of Aldehydes Catalyzed by Platinum Nanoparticles Dispersed in an Amphiphilic Resin. *ACS Catalysis* **2017**, *7*, 7371-7377.

5) Duraczyńska, D.; Serwicka, E. M.; Drelinkiewicz, A.; Socha, R. P.; Zimowska, M.; Lityńska-Dobrzyńska, L.; Bukowska, A. Solvent and substituent effects in hydrogenation of aromatic ketones over Ru/polymer catalyst under very mild conditions. *Molecular Catalysis* **2019**, *470*, 145-151.

6) Matsuda, S.; Masuda, S.; Takano, S.; Ichikuni, N.; Tsukuda, T. Synergistic Effect in Ir- or Pt-Doped Ru Nanoparticles: Catalytic Hydrogenation of Carbonyl Compounds under Ambient Temperature and H2 Pressure. *ACS Catalysis* **2021**, *11*, 10502-10507.

7) Mayer, M.; Wohlgemuth, M.; Salomé Straub, A.; Grätz, S.; Borchardt, L. Hydrogenation of Organic Molecules via Direct Mechanocatalysis. *Angewandte Chemie International Edition* **2025**, *64*, e202424139.
